# Hyperlipidemia-induced cholesterol crystal production by endothelial cells promotes atherogenesis

**DOI:** 10.1038/s41467-017-01186-z

**Published:** 2017-10-24

**Authors:** Yvonne Baumer, Sara McCurdy, Tina M. Weatherby, Nehal N. Mehta, Stefan Halbherr, Pascal Halbherr, Noboru Yamazaki, William A. Boisvert

**Affiliations:** 1 Center for Cardiovascular Research, John A. Burns School of Medicine, University of Hawaii, 651 Ilalo Street, BSB311, Honolulu, HI 96813 USA; 2 Section of Inflammation and Cardiometabolic Diseases, National Heart, Lung and Blood Institute, National Institutes of Health, Bldg 10-CRC, 9000 Rockville Pike, Bethesda, MD 20814 USA; 3 Pacific Biosciences Research Center, Biological Electron Microscope Facility, University of Hawaii, 2538 The Mall, Snyder Hall, Honolulu, HI 96822 USA; 4 InnoMedica Holding AG, Baarerstrasse 34, Zug, 6300 Switzerland; 5 Institute of Fundamental Medicine and Biology, Kazan Federal University, 18 Kremlevskaya Str., Kazan, 420008 Russia

## Abstract

Endothelial cells (EC) play a key role in atherosclerosis. Although EC are in constant contact with low density lipoproteins (LDL), how EC process LDL and whether this influences atherogenesis, is unclear. Here we show that EC take up and metabolize LDL, and when overburdened with intracellular cholesterol, generate cholesterol crystals (CC). The CC are deposited on the basolateral side, and compromise endothelial function. When hyperlipidemic mice are given a high fat diet, CC appear in aortic sinus within 1 week. Treatment with cAMP-enhancing agents, forskolin/rolipram (F/R), mitigates effects of CC on endothelial function by not only improving barrier function, but also inhibiting CC formation both in vitro and in vivo. A proof of principle study using F/R incorporated into liposomes, designed to target inflamed endothelium, shows reduced atherosclerosis and CC formation in *ApoE* ^*−/−*^ mice. Our findings highlight an important mechanism by which EC contribute to atherogenesis under hyperlipidemic conditions.

## Abstract (web summary)

Atherosclerosis is characterized by subendothelial lipid retention believed to be the result of endothelial trancytosis. Here, the authors show that endothelium can take up and process LDL, generating cholesterol crystals that are deposited on the basolateral side of the cells, causing their dysfunction that can be prevented by forskolin/rolipram treatment.

## Introduction

Atherosclerosis is a disease characterized by thickening of the intimal area of the vasculature over many decades. Although the etiology of the disease is complex, it is generally agreed that changes to the endothelium precede early atherosclerosis, characterized by accumulation of lipoprotein particles within the subendothelial space, resulting in perturbation of the protective endothelial monolayer^1^. Under pathological conditions, the endothelial monolayer becomes inflamed and leaky, resulting in uncontrolled trans- and para-cellular transport of cholesterol and water^2, 3^, enhanced monocyte infiltration, and increased endothelial apoptosis with reduced regenerative capacity, leading to atherosclerotic plaque formation over time^4^.

It is well known that EC take up LDL through either receptor-dependent pathways involving caveolae or clathrin-coated pit, or independent of any receptors^2, 5^. However, exactly how the EC process normal LDL, with which they are in constant contact, has not been clearly elucidated, particularly in the in vivo setting. It is also not clear if and how EC respond to normolipidemic versus hyperlipidemic conditions. In the context of atherosclerosis it is generally believed that LDL taken up by EC is transcytosed through the cell and deposited in the intima^6^. During this process, some of the lipoprotein particles become oxidatively modified^3^, causing activation of the overlying EC and subsequent expression of selectins and adhesion molecules that attract monocytes to the area^3^. However, the proportion of lipoproteins that transmigrate to the basolateral side of EC is very small^6^, and how much of it is metabolized by EC themselves remains enigmatic. Emerging studies indicate that uptake of LDL by EC under hypercholesterolemic conditions is facilitated by activin-like kinase 1^7^, and that transcytosis of LDL across EC is mediated by scavenger receptor B1^8^.

Recent studies have demonstrated that cholesterol crystals (CC) may be an important driving force in the pathogenesis of atherosclerosis. Biologically derived CC have been observed using polarized light microscopy (PLM) in advanced atherosclerotic plaques as early as 1959^9^. Crystals within plaque have been reported^10, 11^, but it was only in 1976 that it was unequivocally demonstrated using X-ray diffraction that crystals were derived from cholesterol monohydrate^12^. A recent study using hyperspectral CARS imaging confirmed the presence of crystallized cholesterol in mouse atherosclerotic plaque^13^.

Studies concerning the biological effects of CC within the plaque have focused mainly on the contribution of macrophage foam cells as it is widely believed that CC originate from cholesterol-laden cells in the necrotic core^14^. It has also been shown that under certain circumstances, CC can be formed by active intracellular processing of cholesterol in macrophages^15, 16^. Interest in CC and atherosclerosis was renewed recently as it was shown that CC treatment of macrophages leads to activation of NLRP3-dependent inflammasome activation, driving plaque progression^17–19^. Various factors including temperature, pH, cholesterol hydration and saturation can play a role in CC formation^20^, although the exact mechanism that leads to CC formation has never been uncovered. In particular, the needle-shaped crystals are believed to be especially damaging as they can puncture through the endothelium to weaken plaques, and even cause thrombosis in advanced, vulnerable plaques^21, 22^. Although it is clear that these crystals have pro-inflammatory effects when encountered by macrophages, whether CC are made by EC, particularly during early stages of the disease, has not been explored.

We demonstrate herein that EC are indeed active in processing LDL particles and that CC are formed and deposited subendothelially after only a short period of hyperlipidemia. We further show that the formation of CC and its subsequent pathogenic effects could be inhibited by pharmacologically increasing endothelial cAMP. In addition, administration of liposomes targeting inflamed endothelial cells to deliver cAMP-enhancing agents, decreases atherosclerotic plaque formation and CC content in vivo. On the basis of these findings, we postulate that this subendothelial presence of CC represents a novel and important feature in the pathogenesis of atherosclerosis.

## Results

### ECs generate cholesterol crystals upon LDL treatment

**Fig. 1:**
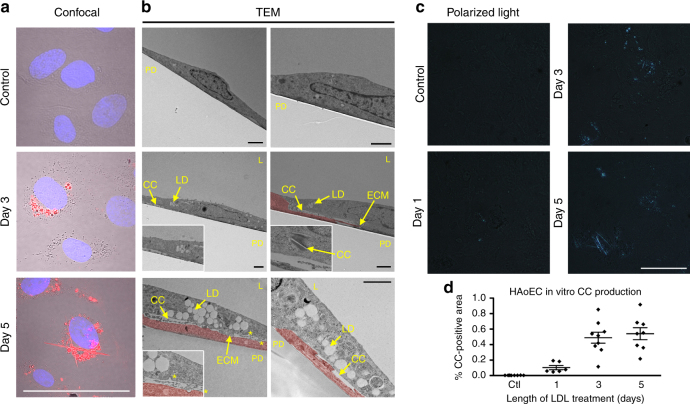
HAoEC produce cholesterol crystals under hyperlipidemic conditions in vitro. Human aortic endothelial cells (HAoEC) were treated with high levels of LDL (250 μg/ml) or DiI-LDL (100 μg/ml) for 3 or 5 days after reaching confluence. The cells were then subjected to confocal microscopy (scale bar = 50 μm) **a**, TEM **b**, or polarized light (PL) microscopy **c**. Quantification of crystalline-positive area after 0-5 days of LDL treatment is shown in **d**. PD: petri dish, L: lumen, CC: cholesterol crystal, LD: lipid droplet, ECM: extracellular matrix, * indicates clathrin-coated pits, red coloring in B indicates area of two overlapping ECs. (scale bar TEM = 500 nm, scale bar PLM = 50μm) (Representative images of *n* = 5, error bar given SEM)

To determine if EC are able to take up and metabolize LDL during hypercholesterolemic state, human aortic endothelial cells (HAoEC) were cultured in vitro and treated with DiI-labeled LDL (Fig. 1a), or unlabeled native LDL (Fig. 1b, c) for 0, 1, 3 or 5 days. Confocal images of HAoEC treated with DiI-LDL using a combination of bright field, DAPI and DiI- imaging revealed modest uptake of LDL at day 3. By day 5, the cells showed signs of robust LDL uptake with prominent lipid droplets visible within the cell. Of particular interest, we observed the presence of DiI-positive needle-shaped particles reminiscent of crystalline structures seen previously in atherosclerotic plaques^13^. This prompted us to further explore the identity of these particles. To determine if the particles were of crystalline nature, LDL-treated HAoEC were viewed using PLM. This technique confirmed irrefutably that the particles were crystals, as indicated by the bright white/blue reflective images seen under PLM, with some appearing to be needle-shaped (Fig. 1c), while untreated control cells did not show the presence of crystals.

Transmission electron microscopy (TEM) performed on 60 nm cross-sections of HAoEC treated with LDL for 3 or 5 days revealed large intracellular lipid droplets (LD), some appearing to be fused (Fig. 1b). We observed ‘clefts’ characteristic of the space that crystalline particles leave after tissue processing with organic solvents, which we have labeled cholesterol crystals (CC). LDL treatment for 3–5 days led to increased EC overlaps (colored), with atypical vacuous spaces between adjacent cells. In addition, these overlaps were filled with extracellular matrix (ECM) components and clefts, indicating the presence of CC both intracellularly and on the basolateral side of the EC monolayer. In contrast, HAoEC left untreated remained in a tightly connected, confluent monolayer displaying almost no LD or clefts. These in vitro results demonstrate that (1) HAoEC are capable of robust LDL uptake, and (2) that ECs produce CC under hyperlipidemic conditions, implicating a possible role of EC-mediated CC formation in the earliest stages of atherogenesis.

To test whether other cell types of human origin are capable of CC formation, we treated monocyte-derived macrophages (HMDM), skin fibroblasts (HSF), umbilical vein endothelial cells (HUVEC) and smooth muscle cells, in addition to HAoEC, with low and high doses of LDL, OxLDL and AcLDL (Supplementary Fig. 1a–d). Among all of these cells and conditions, only LDL-treated HAoEC showed an appreciable amount of CC formation (Supplementary Fig. 1d). Inset panels for each cell type show F-actin staining with Phalloidin (green) revealing healthy cell morphology.

The presence of CC in the neointima of human atherosclerotic plaque was examined in aorta and femoral artery tissue samples from the same patient post-mortem. The early plaque seen in the aorta sections contained large amounts of CC as seen by PLM, whereas femoral artery sections with no visible atherosclerosis showed no CC (Supplementary Fig. 2a). Analysis of these tissue samples via TEM revealed numerous clefts in aorta samples whereas very few were seen in the femoral artery sections (Supplementary Fig. 2b).

### CC formation occurs in early atherosclerosis

**Fig. 2:**
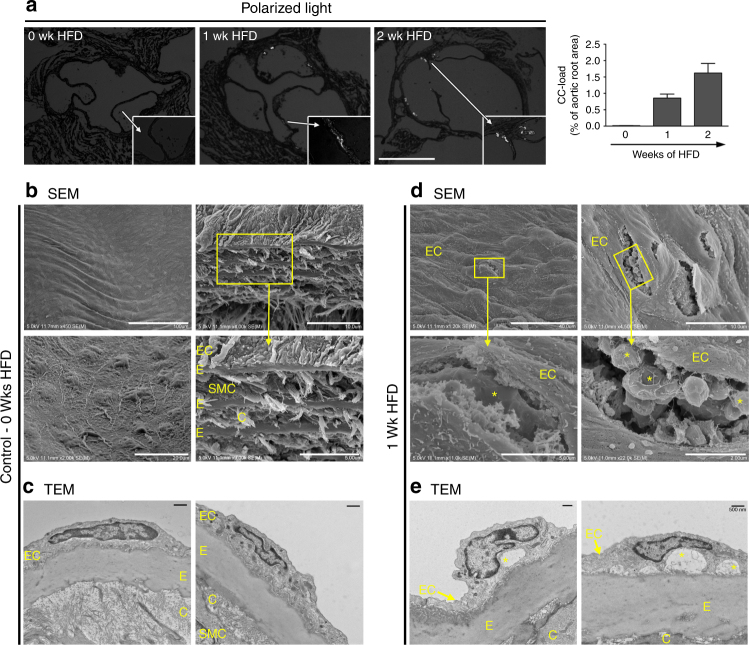
Subendothelial cholesterol crystals are found after 1-week of HFD in vivo. **a** Aortic sinus sections from *Ldlr* ^*−/−*^ mice on HFD were subjected to PLM to quantify the CC load. (representative images of *n* = 4, error bars represent SEM, scale bar = 500 μm) **b**–**e** The first CC deposition beneath the endothelial monolayer is observed within 1 week of HFD and increases over time. (representative images of *n* = 5) **b** The thoracic aorta and aortic arch of *Ldlr* ^*−/−*^ mice were subjected to SEM or TEM respectively. SEM images of control mouse aorta surfaces display a confluent, smooth endothelium overlaying the vessel surface followed by alternating layers of elastin (E), as well as smooth muscles cells (SMC) and collagen (C). **c** TEM images of a representative aorta cross-section from a control *Ldlr* ^*−/−*^ mouse displays a continuous endothelial monolayer in close contact with the underlying elastin layer. **d** Following 1 week of HFD treatment, subendothelial protrusions from the aortic surface are visible by SEM. Openings in these aortic surfaces (boxes) and the enlarged view in the lower panels display the deposition of subendothelial, solid crystalline particles of various shapes and sizes (*) embedded in ECM. **e** TEM analysis of 1-week HFD aortas display subendothelial deposition of particles, which appear as “clefts” (*), covered by a continuous EC layer (scale bar TEM = 500 nm)

Although it is well established that CC are present in advanced atherosclerotic plaques^23^, our results indicate that under hypercholesterolemic conditions, HAoEC produce CC in vitro just days after encountering and processing native LDL, leading us to question whether CC formation might occur in early atherosclerosis development. To estimate the timeline of CC appearance in the developing plaque, we analyzed aortic root sections of *Ldlr* ^*−/−*^ mice fed a high cholesterol/high fat diet (HFD) for 0–2 weeks using PLM (Fig. 2a). Interestingly, the first signs of CC in the plaque occurred within only 1 week of HFD, before signs of macrophage infiltration or neointima formation. Control *Ldlr* ^*−/−*^ mice on normal chow did not contain any CC, whereas CC content was increased with prolonged length of HFD treatment (graph in Fig. 2a). Our data are in agreement with the findings of Duewell et al., who describe the presence of CC in *ApoE* ^*−/−*^ mouse plaque after 2 weeks of HFD^19^.

To further confirm the presence of early subendothelial CC formation in vivo, we analyzed the aortic arch of control (no HFD) and 1-week HFD-fed *Ldlr* ^*−/−*^ mice using scanning electron microscopy (SEM) and TEM (Fig. 2b–e). The surfaces of control aortas were smooth with a continuous layer of tightly connected EC (Fig. 2b). Openings in the surface of the vessel (Fig. 2b), a result of the preparation process, demonstrate the anatomy of a normal blood vessel, with a thin endothelial monolayer in tight contact with elastin fibers (E) followed by alternating layers of smooth muscle cells (SMC)/collagen (C) and elastin layers. After only 1 week of HFD, however, dramatic changes were observed (Fig. 2d), with the endothelial layer displaying prominent protrusions from underlying cholesterol deposits. Openings in the luminal surface (Fig. 2d) allow us to observe the subendothelial side of the plaque, where solid, smooth particles are present just below the endothelial monolayer.

TEM sections of control aortas (Fig. 2c) revealed a tight connection between EC and the underlying elastin layer with no subendothelial deposition of particles. In contrast, after 1-week of HFD, large subendothelial deposits can be seen (*) (Fig. 2e), which also correlate with the particles seen beneath the EC layer in SEM images from the same aortas (Fig. 2d). Although the exact mechanism is not yet clear, we believe that the CC nucleate within EC, are secreted to the subendothelial space, and grow in size as more lipid and crystals are deposited.

### Altering cholesterol metabolism affects CC formation

**Fig. 3:**
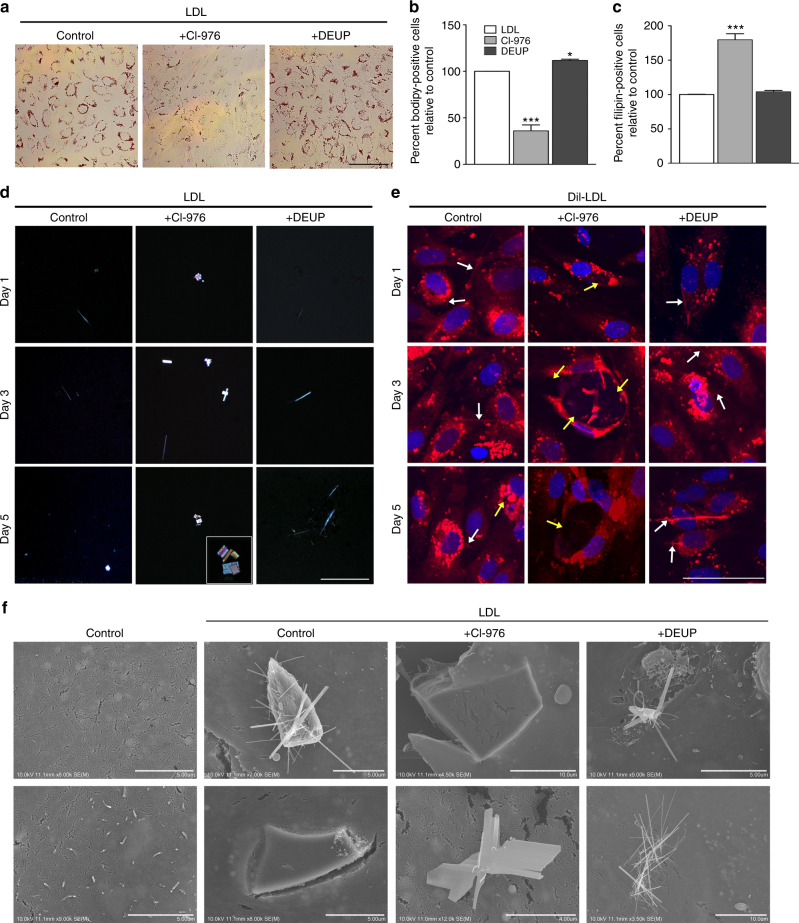
Cholesterol crystal production affected by altering FC-CE abundance. **a**–**c** HAoEC were treated for 3 days with 250 μg/ml LDL alone, or in parallel with CI-976 to inhibit the cholesterol ester transferase ACAT, or the cholesterol ester hydrolase DEUP to increase free cholesterol (FC) or cholesteryl ester (CE) content respectively. **a** Staining of neutral lipids with Oil Red O after 3 days of LDL treatment reveals differences in cellular CE content (scale bar = 100 μm). **b, c** EC were also analyzed by flow cytometry after being stained with Bodipy to detect CE **b**, or Filipin to detect FC (**c**) (*n* = 3 in duplicates, error bar indicates SEM, * indicates significance to LDL treatment at *p* < 0.05, *** *p* < 0.001 in unpaired t-test). **d** Polarized light microscopy analysis shows an increase in large, plate-shaped crystals after CI treatment, while DEUP treatment results in increased abundance of needle-shaped crystals as compared with LDL-treated control HAoEC. (scale bar = 50 μm) **e** Treatment of HAoEC with DiI-LDL for 1, 3 or 5 days reveals similar uptake of LDL between groups, although more needle-shaped CC (white arrows) are seen in DEUP-treated cells, while large DiI-negative spaces (yellow arrows) that we believe may be crystalline particles were observed in CI-976-treated cells. (scale bar = 50 μm) **f** SEM images of HAoEC in vitro after 5 days of LDL treatment reveal production of cholesterol crystals, with CI-976 treatment leading to mainly plate-shaped and DEUP treatment leading to mainly needle-shaped CC. (representative images of *n* = 4)

Next, we sought to determine whether the storage form of cholesterol, cholesteryl ester, or free cholesterol (FC) contributed more to CC formation in EC. To study this, we utilized two well-known inhibitors; ACAT inhibitor CI-976 (raises FC) and CE hydrolase inhibitor DEUP (raises CE). By treating HAoEC with these inhibitors, we verified through Oil Red O, Bodipy and filipin staining, that CI-976 indeed raises FC levels and DEUP raises CE levels (Fig. 3a–c). Treatment of cells with DiI-LDL and CI-976 dramatically increased the formation of CC by EC, whereas treatment with DEUP showed a modest increase of CC with propensity of the crystals to be more needle-shaped as shown by PLM (Fig. 3d). DiI-LDL uptake was similar among the 3 groups (Fig. 3e), indicating that differences in CC formation were not due to differential LDL uptake. DiI-LDL treated HAoEC, especially those co-treated with DEUP, showed the presence of thin, DiI-positive, needle-shaped structures (Fig. 3e, white arrows) that correlate with the needle-shaped crystals observed by PLM. Cells treated with DiI-LDL and CI-976 show large DiI-negative spaces (Fig. 3e, yellow arrows), that we believe may represent plate-shaped crystals that were formed due to the increase in FC processed from the DiI-LDL. Clear SEM examples of both plate- and needle-shaped crystals, induced by treatment with CI-976 or DEUP, respectively, are shown in Fig. 3f. Control cells without inhibitor treatment tended to show both shapes of crystals.

### Effects of cholesterol crystals on endothelial function

We next explored the effects of subendothelial CC on endothelial function in vitro. For this we devised a culture system in which the EC monolayer was cultured on top of synthetic CC embedded in gelatin. This was experimentally necessary because by the time CC were formed by EC, the cells were no longer healthy enough to be used for further experiments. Synthetic CC were assessed by SEM and found to have various sizes and shapes (Supplementary Fig. 3a). Using energy-dispersive X-ray spectroscopy (EDS) analysis of both synthetic and naturally-occurring CC samples, we verified the organic carbon-based chemical composition of synthetic CC to be remarkably similar to CC found in vivo after 1 week of HFD (Supplementary Fig. 3b, c). CC were visible via PLM only in cells with embedded CC (Supplementary Fig. 3d, e). In addition, EC cultured on synthetic CC showed lipid droplet accumulation, indicating active processing of the gelatin-embedded CC (Supplementary Fig. 3f).

**Fig. 4:**
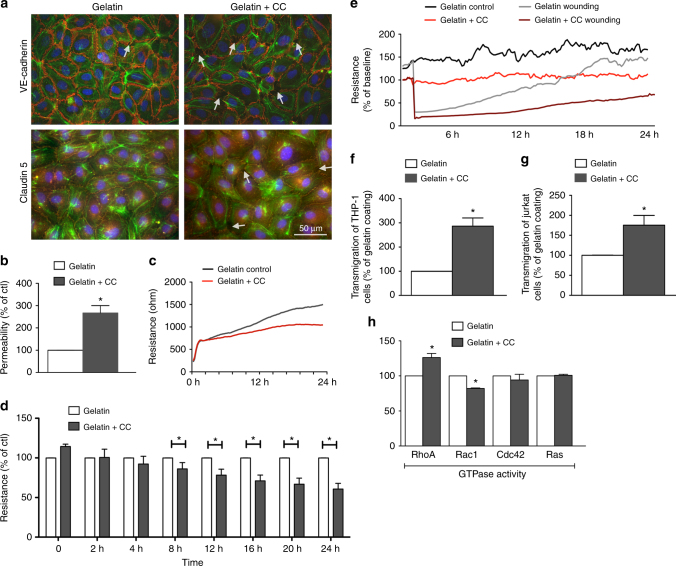
HAoEC become dysfunctional when cultured on cholesterol crystals in vitro. HAoEC were cultured on gelatin or gelatin+CC and kept in culture for 5 days until confluent. **a** HAoECs cultured on gelatin resulted in formation of a confluent endothelial monolayer within 5 days. VE-cadherin and claudin-5 distribution (both shown in red) along the cell borders as well as moderate actin stress fiber formation (green) are shown by immunofluorescence staining. HAoECs grown to confluence on gelatin+CC resulted in numerous gaps between cells (arrows) visualized by VE-cadherin and claudin-5 staining, indicating incomplete junction formation. No changes in actin distribution were observed. (representative images of *n* = 3, scale bar = 50 μm) **b** A transwell permeability assay was used to determine endothelial barrier function. HAoEC grown on gelatin + CC vs. gelatin alone show 2.5-fold increased permeability (*n* = 3 in triplicate). Using ECIS, endothelial barrier integrity was determined **c**, **d**. An exemplary TER curve for cells inoculated on gelatin or gelatin + CC coated electrodes **c**, and data from 5 experiments are summarized **d**. HAoEC grown on CC show nearly 40% reduced TER compared with cells grown on gelatin. **e** Using the ECIS wounding assay, the regenerative potential of HAoEC was analyzed and TER over 24 h post-wounding is shown as a percentage relative to gelatin+CC. HAoEC cultured on gelatin display full recovery within 24 h, while cells grown on gelatin+CC display delayed regeneration and incomplete recovery of barrier integrity. **f**, **g** Transwell filter assays were used to determine trans-endothelial migration of THP-1 and Jurkat cells towards the chemoattractants MCP-1 and SDF-1 respectively. Increased trans-endothelial passage of both cell types was observed when HAoEC were cultured on gelatin+CC transwell filters (*n* = 5 in duplicates). **h** GLISA assays show alterations in activity of Rho-GTPases after cultivation on gelatin+CC vs. gelatin alone. RhoA activity increased while Rac1 activity decreased. Cdc42 and Ras activity were not affected. (*n* = 5) (Error bars represent SEM, *p < 0.05)

Endothelial barrier integrity was significantly compromised when EC were cultured on CC (Fig. 4) as evidenced by disrupted VE-cadherin and claudin 5 staining (red), and actin stress fiber formation (green) indicating impaired endothelial junction formation (Fig. 4a). A transwell permeability assay as well as electric cell-substrate impedance sensing (ECIS) was used to quantitatively measure endothelial integrity. Subendothelial CC resulted in a 2.5-fold increase in permeability and a 30% decrease in resistance compared with control cells (Fig. 4b–d). Endothelial regeneration, evaluated using the ECIS wounding system, was also compromised (Fig. 4e). To test if this compromise in barrier integrity would affect the transendothelial migration of monocytes and T-cells, HAoEC were cultured with or without CC in a transwell filter setup. When subendothelial CC were present, we observed approximately 3-fold and 2-fold increases in migration of THP-1 and Jurkat cells, respectively (Fig. 4f, g). These data indicate that early subendothelial CC deposition leads to impaired endothelial integrity and may facilitate the migration and subsequent accumulation of leukocytes, which are known to promote atherosclerosis.

As previous studies have shown that Rho-GTPases are major regulators of endothelial barrier function^24^, we questioned whether early CC deposition may have an effect on endothelial function via alteration of Rho-GTPase activity. While Ras and Cdc42 were not altered as a result of subendothelial CC deposition, RhoA activity was enhanced and Rac1 activity was suppressed (both known to be associated with endothelial dysfunction) (Fig. 4h), indicating that the observed effects could at least partially be driven by alteration of RhoA/Rac1 activity. Despite RhoA’s involvement, inhibiting its well-known effector, Rho-kinase, did not affect EC function (Supplementary Fig. 4).

### F/R mitigates CC-mediated endothelial dysfunction

**Fig. 5:**
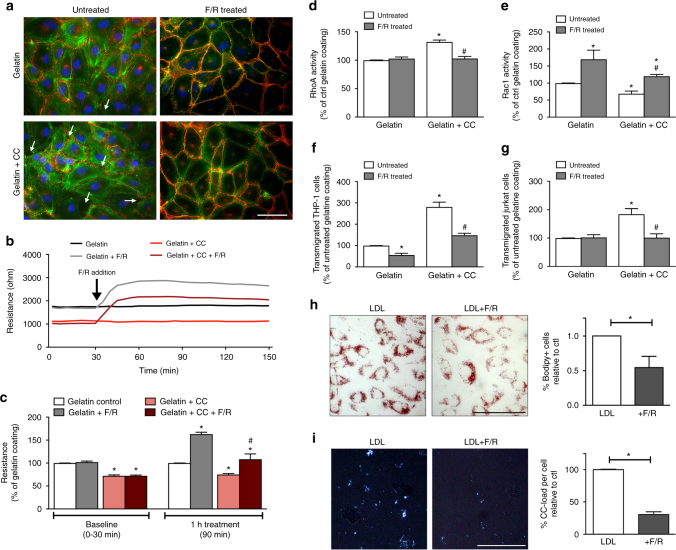
F/R rescues CC-induced endothelial dysfunction in vitro. **a**–**e** HAoEC were cultured on gelatin or gelatin + CC and either left untreated, or were treated with 10 μM Forskolin/5 μM Rolipram (F/R). (representative images of *n* = 5) **a** Staining of HAoECs for VE-cadherin (red) and actin cytoskeleton (green) is shown by immunofluorescence. Stronger VE-cadherin staining, as well as decreased stress fiber formation and increased cortical actin is observed in F/R-treated cells. Gap formation (arrows) induced by CC was completely eliminated after F/R application. (scale bar = 50 μm) **b** A representative barrier integrity curve for HAoEC cultured on gelatin or gelatin+CC with F/R treatment is shown and quantified in **c** as percentage of gelatin control at each time point. The application of F/R increases resistance for HAoEC grown on gelatin as well as CC surfaces (*n* = 5). **d**, **e** HAoEC grown on gelatin and treated with F/R showed increased Rac1 activity and no changes in RhoA. ECs grown on gelatin+CC and treated with F/R show inhibition of the previously observed CC-induced RhoA activation and reversal of the CC-induced Rac1 inactivation above control levels (*n* = 5). **f**, **g** Transwell filter migration assays revealed increased transendothelial migration of THP-1, as well as Jurkat cells when HAoEC are cultured on CC. F/R treatment reverses this effect. (*n* = 5). **h**, **i** HAoEC were treated with LDL or LDL+F/R for 3 days. (*n* = 4) **h** Cells were stained with Oil Red O and imaged, or stained with Bodipy and analyzed by flow cytometry to measure cholesteryl ester (CE) content. (scale bar = 100 μm) **i** Cells were analyzed by PL microscopy to visualize and measure CC content. (Error bars represent SEM, *p < 0.05, **c**–**i**: *p < 0.05 to gelatin ctl; #*p* < 0.05 to gelatin+CC ctl)

As compromised endothelial barrier integrity caused by subendothelial CC could have a significant impact on atherogenesis, we explored means by which CC-mediated endothelial barrier destabilization could be minimized. Our group and others have shown previously that treating EC with forskolin, an adenylate cyclase activator, and rolipram, a phosphodiesterase IV inhibitor, can strengthen endothelial barrier^25, 26^. Culturing HAoEC with CC resulted in formation of gaps between cells in the endothelial layer, as visualized by staining of VE-cadherin (red) and actin cytoskeleton (green), which was abrogated by treatment with F/R (Fig. 5a). ECIS was used to determine if barrier integrity compromised by CC could also be rescued by F/R. Differences in resistance appeared within minutes in both control and CC-coated cells following F/R treatment (Fig. 5b, c). F/R administration also led to lower RhoA and higher Rac1 activity (Fig. 5d, e), another indication of improved endothelial integrity. Moreover, treatment with F/R abrogated the CC-induced increase in transendothelial migration of monocytes and T-cells (Fig. 5f, g). When HAoEC were incubated with LDL±F/R, the cells treated with F/R showed significantly less lipid loading (Fig. 5h), as well as decreased CC presence (Fig. 5i).

### F/R decreases subendothelial CC deposition in vivo

**Fig. 6:**
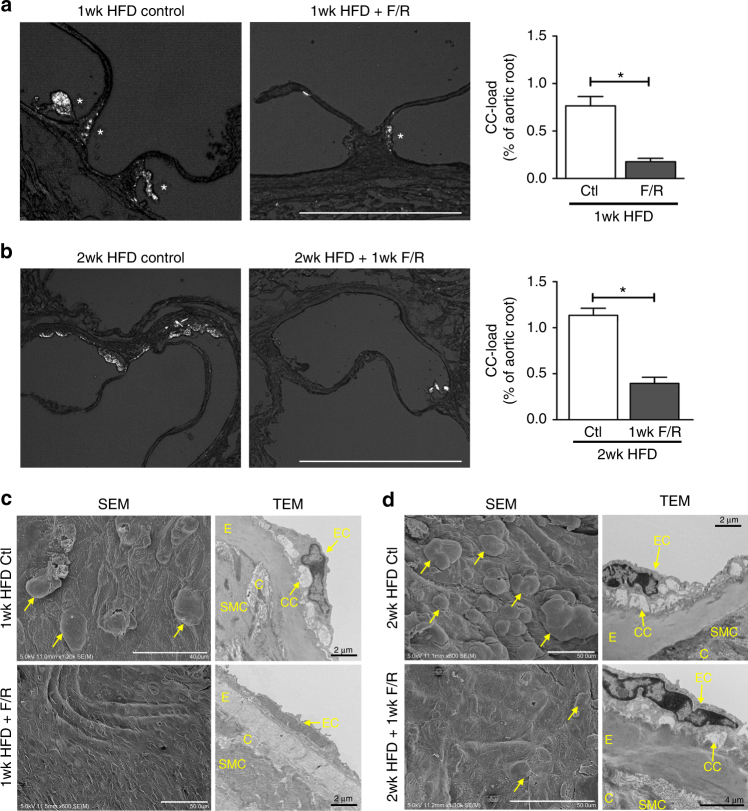
F/R rescues subendothelial CC production in vivo. **a**, **c** *Ldlr* ^*−/−*^ mice were fed a HFD for 1 week with simultaneous IP injections with F/R or PBS (control) every other day. **b**, **d** *Ldlr* ^*−/−*^ mice were kept on HFD for 1 week before initiation of IP injections with F/R or PBS (control) every other day for an additional week on HFD. **a**, **b** Aortic root sections were subjected to PL to visualize CC. (scale bar = 500 μm) **c**, **d** The aortic arch was used for analysis by SEM (luminal surface) and TEM. Yellow arrows show subendothelial protrusions from the aortic surface. (Error bars represent SEM, *p < 0.05, representative images of *n* = 8, IP: intraperitoneal injection, C: collagen, CC: cholesterol crystal, SMC: smooth muscle cell, E: elastin layer, EC: endothelial cell)

To assess whether these protective effects of F/R treatment on HAoEC could be recapitulated in vivo, 4 groups of *Ldlr* ^*−/−*^ mice were placed on HFD. Two of the groups were fed HFD for 1 week, one group receiving treatment with F/R, and the control group receiving PBS via IP (intraperitoneal) injections. The other 2 groups were given the HFD for 1 week, followed by another week of HFD with PBS or F/R IP injection, to test whether early plaque development could be abrogated by F/R treatment once the mice had already been hyperlipidemic for a week. In both cases, the mice treated with F/R showed significantly reduced CC load in the aortic sinus plaque shown by PLM (Fig. 6a, b). SEM and TEM photos also revealed smoother endothelial surface and less CC deposition in the subendothelial space (Fig. 6c, d).

### Targeted F/R delivery to inflamed endothelium in vivo

Despite its effectiveness in reducing CC formation, F/R injection into mice resulted in acute fatigue of the animals, most likely due to a dramatic lowering of blood pressure. These side effects and the narrow therapeutic window rendered the use of F/R as an experimental anti-atherosclerotic agent unfeasible. After exploring several options we decided to use a targeted drug delivery system with liposomes from our collaborators at InnoMedica to specifically target inflamed endothelium. This enabled us to conduct a long-term animal study to further investigate the beneficial effects of F/R on CC production and more advanced atherosclerosis development. To assess the best target for liposome delivery, we incubated HAoEC with LDL for 6, 12, or 24 hours, and then measured whether the three most common endothelial adhesion molecules (ICAM-1, VCAM-1, E-Selectin), were upregulated by LDL. Only E-selectin surface expression was consistently upregulated (Supplementary Fig. 5a), even though all three were found to be upregulated at the transcript level (Supplementary Fig. 5b). Furthermore, we verified that HFD increases surface expression of E-selectin in vivo using flow cytometry (Supplementary Fig. 5c). Based on these results we chose to conjugate the liposomes with the well-known ligand of E-selectin, Sialyl Lewis X (sLe^x^) sugar chains, to facilitate their binding to E-selectin (Supplementary Fig. 6a). E-selectin is a well-established endothelial inflammatory marker which is robustly expressed on atherosclerosis-prone endothelium^27^. TEM analysis was used to calculate the size of the liposomes, which ranged from 76 to 150 nm with a median size of 121 nm (Supplementary Fig. 6b).

To characterize the binding of liposomes in vitro, as well as to empirically determine the concentrations to use for further experiments, HAoEC were treated with various dilutions of DiI-liposomes in the absence or presence of TNFα for 24 h. Cells were subsequently analyzed using fluorescence microscopy and flow cytometry (Supplementary Fig. 6c, d). Significant uptake of liposomes was observed using dilutions of 1:50 to 1:25, which became more prominent with the addition of TNFα, a known inducer of E-selectin on the endothelial surface. The optimum dosage was calculated to be 1:25 dilution of liposomes in vitro and 200 μl of liposome suspension for in vivo experiments.

**Fig. 7:**
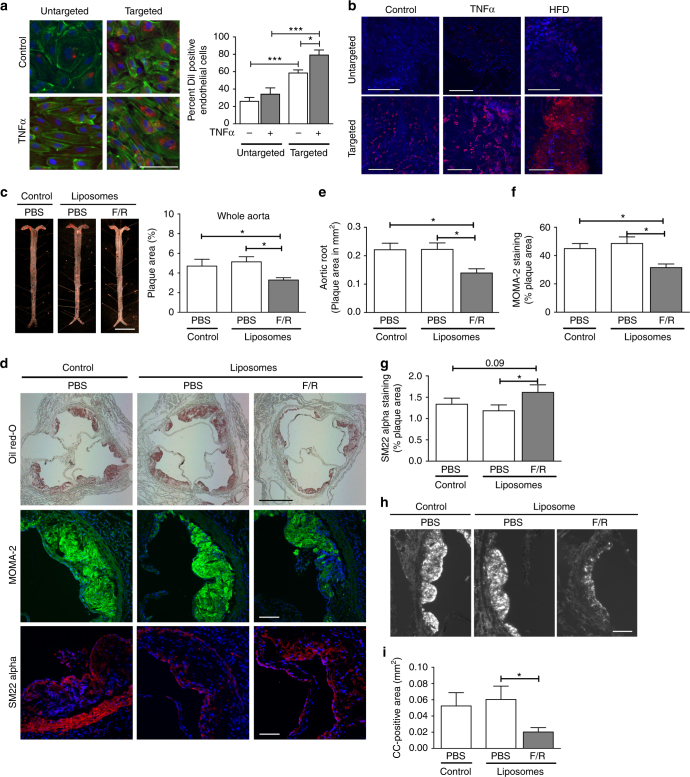
Targeted delivery of F/R-liposomes to inflamed endothelium reduces atherosclerosis development in vivo. **a** HAoEC treated with untargeted or targeted DiI-filled liposomes were analyzed by fluorescence microscopy and flow cytometry. (representative images of *n* = 4, scale bar = 50 μm) **b** After IP injection with PBS or 10 ng/ml TNFα 24 h before killing or 1 week HFD, *ApoE* ^*−/−*^ mice were treated with DiI-filled untargeted or sLe^x^-targeted liposomes via tail vein injection. Aortas were collected and analyzed by fluorescence microscopy for areas of DiI-liposome uptake. (Scale bar = 100 μm, representative images of *n* = 3) **c**–**i** *ApoE* ^*−/−*^ mice were fed a HFD for 6 weeks while receiving weekly IP injections of PBS, or tail vein injection of targeted liposomes filled with either PBS or 10 μM Forskolin/5 μM Rolipram (F/R). (representative images of *n* = 8) **c** Aortas were stained with Oil Red O and plaque areas were quantified with ImageJ software. **d** Aortic root sections were stained using Oil Red O to visualize plaque development, MOMA-2 to visualize macrophage content, SM22α to visualize SMC content, and PL to visualize the CC formation **h**. These values were quantified in **e**, **f**, **g**, and **i**, respectively. (Error bars represent SEM, *p < 0.05, IP: intraperitoneal, scale bar Aorta = 1 cm, Oil Red-O = 500 μm, scale bar MOMA-2/SMA/PL = 100 μm, IP intraperitoneal injection)

To test the specificity of liposome binding to HAoEC, both untargeted and targeted DiI-liposomes were tested in vitro. Without TNFα treatment, 60% of the HAoEC were DiI-positive, which increased to 80% with TNFα treatment, compared with 25% and 30%, respectively, using untargeted liposomes (Fig. 7a). After TNFα treatment, HAoEC uptake of targeted liposomes loaded with F/R resulted in a 4-fold increase in intracellular cAMP levels, a known effect of F/R^28^ (Supplementary Fig. 6e), providing evidence of successful drug delivery to HAoEC.

To investigate the uptake of liposomes in vivo, *ApoE* ^*−/−*^ mice were fed a HFD for 1 week or IP injected with TNFα 24 h before killing to induce inflammation, while control mice received PBS injections. The mice were also treated with 200 μl of targeted or untargeted DiI-liposomes via tail vein injection 24 h before killing. After killing, the inner surfaces of the aortic arches were subjected to laser scanning confocal microscopy to visualize DiI-positive cells (Fig. 7b). Only trace amounts of untargeted DiI-liposomes were bound to the aortic surface after TNFα or HFD treatment, while no DiI was visible in control aortas. In contrast, the sLe^x^-conjugated DiI-liposomes were successfully delivered to the endothelial cells of control, TNFα- and HFD-treated aortas, in increasing order. The presence of DiI was especially prominent at areas of disturbed flow in the aortic arch, the most susceptible area to plaque development. These positive results indicated that this innovative drug delivery system could be used to administer F/R specifically to inflamed endothelial cells during a long-term atherosclerosis study.

### Effect of F/R-liposomes on atherosclerosis in *ApoE*^*−/−*^ mice

To test the effectiveness of our targeted F/R-containing liposome treatment in reducing atherosclerosis burden, we performed a proof of principle study in *ApoE* ^*−/−*^ mice with three treatment groups: (a) PBS, (b) PBS-filled targeted liposomes, or (c) F/R-filled targeted liposomes via tail vein injection once per week while being fed a HFD for 6 weeks. Upon completion of the study, analysis of atherosclerotic plaque area in the whole aorta revealed that treatment with PBS-liposomes did not affect atherosclerosis development (Fig. 7c), whereas animals treated with F/R-liposomes displayed a significant reduction in atherosclerosis burden compared with control mice (Fig. 7c) Analysis of aortic root sections (Fig. 7d) confirmed that atherosclerotic plaque development was significantly decreased only in mice treated with F/R-liposomes (Fig. 7e). Immunofluorescent staining of aortic root sections for the presence of macrophages and SMC (Fig. 7d) revealed a decrease in macrophage content and an increase in SMC content (Fig. 7f, g) in F/R-liposome-treated mice compared with controls, whereas no differences were observed in necrotic core area (Supplementary Fig. 7a). Importantly, analysis of CC in the plaques of the mice revealed that F/R-liposome treatment led to a significant reduction in CC content of the plaque compared with both control-PBS- and PBS-liposome-treated mice (Fig. 7h). There were no significant differences in physiological measurements among the study groups, including body weight, cholesterol and triglyceride levels, as well as circulating leukocyte populations (Supplementary Fig. 7b–d).

## Discussion

Although EC are in constant contact with circulating lipoproteins, whether these cells take up and metabolize the lipoprotein particles has not been well characterized. Our study provides new evidence that EC not only take up and metabolize lipoproteins, but when they are burdened with excess intracellular cholesterol that they generate CC. To our knowledge, we are the first to report the synthesis of CC by EC treated only with LDL, and to show the presence of CC in subendothelial space of *Ldlr* ^*−/−*^ aortas after only 1 week of HFD when macrophages are largely absent. The appearance of CC in plaque is commonly associated with macrophages in advanced atherosclerosis. We believe that this is a source of CC in the plaque that is quite distinct from the initial production of CC by EC in the initial stage of atherosclerosis development, as we have shown in the current study. CC have been reported to be present in atherosclerotic lesions of *ApoE* ^*−/−*^ mice fed the HFD for 2 weeks, however their appearance occurs in parallel with infiltrated inflammatory cells, which continues with increasing length of HFD^19^. As dendritic cells (DC) have also been seen in atherosclerotic plaques^29–31^, and these cells are known to take up lipids soon after induction of hypercholesterolemia^32^, it is possible that the DCs contributed to the formation of CC in our mice. However, we found very few DC in our 1–2 week HDF-fed aortas of both *ApoE* ^*−/−*^ and *Ldlr* ^*−/−*^ mice (Supplementary Fig. 8). Furthermore, the majority of the CC that we observed in the aorta samples of early atherosclerosis were not associated with myeloid cells (macrophages and/or DC), indicating that the CC formation in early plaques is independent of myeloid cell presence.

The importance of CC in atherosclerosis was established nearly 40 years ago when a comprehensive study examining the lipid composition of human plaques found that CC are present in advanced stages of atherosclerosis^12, 21^. Prevailing current view of CC in the context of atherosclerosis is that crystals form as a result of lipid saturation originating from subendothelial macrophage foam cells. Studies using macrophages have reported CC production upon OxLDL treatment^15^ or cholesteryl ester lipid droplets^16^, but not with native LDL treatment. We chose to use LDL in our studies because in normal physiological conditions EC do not encounter modified LDL. Using *Ldlr* ^*−/−*^ animals for our in vivo studies did not interfere with endothelial LDL uptake since most of the LDL taken up by EC occurs through receptor-independent endocytosis^6^. Moreover, CC formation is not a result of cells dying since treating HUVEC and HSF with LDL, OxLDL or AcLDL resulted in high rate of cell death, but little to no CC formation.

Although there are certain physical parameters that are known to affect CC formation and/or growth such as temperature, hydration and pH^20^, any information pertaining to biological mechanism(s) that contribute to CC formation are very scant, especially for EC. Based on our observations, we can speculate that when EC encounter high levels of LDL, lipoproteins are taken up and processed intracellularly. The excess cholesterol is esterified with the help of ACAT and stored in lipid droplets (LD). When cells are overloaded with LD, we believe that the equilibrium shifts and EC try to break down LD by activating the lysosomal pathway involving the lysosomal acid lipase which converts the CE to FC. In other cell types like the macrophage, the FC can be excreted via the cholesterol efflux pathway utilizing the cholesterol transporters *ABCA1* and *ABCG1*, which we have shown previously to be highly upregulated in lipid-loaded macrophages^33^. In EC, however, we have found that *ABCA1* and *ABCG1* are not upregulated even after the cells have taken up LDL (Supplementary Fig. 8e), indicating that cholesterol efflux in lipid-laden EC is not a robust mechanism. Thus, lipids accumulate in the cell and become crystallized, although the exact mechanisms involved in the cholesterol crystallization process needs to be explored.

It is also possible, according to our findings, that liquid crystals, also known as anisotropic droplets or spherulites, are produced in the EC as a result of LDL uptake and processing. These premature crystals may be secreted to the basolateral side of the endothelium, subsequently coated with ECM and grow into crystals by fusing with other crystals and possibly free lipid droplets.

As an alternative view to explain cellular lipid degradation, more recent studies have found that intracellular lipid metabolism is mediated by lipophagy involving autophagosomes and lysosomes and the fusion of these two organelles to degrade cellular lipid stores^34^. If lipid degradation via lipophagy occurs in EC, we can assume that the autophagosomes fuse with lysosomes, leading to lysosomal FC generation. In support of this, it was reported recently that cell-based CC formation occurs mostly in acidic lysosomes^15^. However, although LDL degradation may occur through the autophagosome-lysosome-lipophagy pathway, the mechanism to excrete the processed cholesterol, whether in lipid droplet, liquid crystal or crystal form, to the basolateral side is unknown. One possible mode of intracellular transport is through multi-vesicular bodies (MVB) that are found commonly within EC. While CC may form in acidic lysosomes, a recent study suggested that most of the cholesterol in the endocytic pathway is harbored in MVB^35^. We postulate that the initial CC formation in EC occurs in TSG101-positive MVB. TSG101 is necessary for MVB formation and vesicular trafficking as part of the endosomal sorting complex required for transport (ESCRT) machinery, and a key marker of MVB^36^. According to a recent report, the storage and metabolism of lipids in EC, especially free cholesterol, occurs in vesicular structures which are surrounded by membranes rich in free cholesterol as well as caveolin-1^37^. Thus, while more studies are clearly needed to fully elucidate the mechanisms involved in CC formation and transport, it appears that they require the orchestration of multiple cellular processes.

Interestingly, it was shown in human atherosclerotic samples that changes in serum lipid composition (via statin treatment) can alter the appearance and shape of CC^38^. Needle-shaped crystals are most commonly reported in atherosclerotic plaques^22^, although CC in more advanced atherosclerotic plaques can be plate-shaped^12, 13, 39^ or random aggregates forming rod-like structures as well^40^. It is noteworthy that electron microscopy images of CC published by these groups closely match the plate and needle-shaped CC that we observed in our experiments. We believe that the initial crystals found in the subendothelial space in fact are produced by EC and attract monocytes to transmigrate into this space. Once these monocytes differentiate into macrophages they take up CC as well as other forms of free lipids and perpetuate the formation and progression of atherosclerotic plaque. Interestingly, it was reported recently that a C-type lectin receptor called Mincle can bind to CC and trigger a pro-inflammatory immune response in human macrophages as well as dendritic cells^41^. This was the first demonstration of a direct innate immune receptor for CC, but it remains to be seen whether CC-Mincle interaction is an active phenomenon in atherosclerosis.

Although the mechanism behind F/R’s ability to reduce CC formation under hypercholesterolemic conditions, and thereby attenuate the atherosclerotic burden, remains to be investigated, forskolin and/or rolipram used as therapeutic agents have proven to be beneficial in a number of conditions including spinal muscular dystrophy^42^, septic shock^43^, rheumatoid arthritis^44^, memory performance^45^ and pulmonary capillary ischemia-reperfusion injury^46^. Importantly, recent research suggests that administration of F/R results in stimulation of lipolysis and attenuation of weight gain in rats fed a HFD^28^. The sLe^x^-liposomes used in our studies were prepared exclusively for this study and were meant to demonstrate the delivery of F/R to inflamed endothelium as a proof-of-principle only. It is worth noting, however, that the utility of this liposome system has been demonstrated by other investigators in a variety of disease models. For example, the sLe^x^-conjugated liposomes loaded with fluorescent material designed to target tumors have been utilized for in vivo imaging of tumors^47^. Furthermore, sLe^x^-liposomes have been used successfully to deliver dexamethasone to inflamed eyes in a murine model of experimental autoimmune uveoretinitis^48^. Most recently, sLe^x^-liposomes containing doxorubicin along with several controls were injected into rats that have undergone angioplasty to prevent stenosis. The rats that received the drug incorporated into the sLe^x^-liposome exhibited significantly larger lumen area compared with controls^49^. These studies along with our own data on the use of sLe^x^-liposome indicate that E-selectin expressed on inflamed endothelium is a useful target to deliver drugs and other agents to the affected endothelium, including atherosclerosis-prone areas.

In summary, we show in vitro that aortic endothelial cells produce CC under conditions of hyperlipidemia, and that CC accumulate subendothelially in vivo after only 1 week of HFD, a time point that precedes macrophage infiltration. This phenomenon can be prevented by F/R administration as demonstrated by a study in which short-term use of F/R in vivo successfully inhibited endothelial CC production after 1 week of HFD. Furthermore, we report an effective method of specific drug delivery to inflamed endothelium using F/R-incorporated liposomes conjugated with sLe^x^. This technique is particularly useful when the systemic application of a drug results in adverse side effects, as is the case with F/R. Using this specialized delivery system, we have verified the specific targeting of inflamed endothelial cells and clearly demonstrated that this application reduces the development of atherosclerosis in vivo.

## Methods

### Materials

Human LDL, DiI-LDL, oxLDL and AcLDL were purchased from Alfa Aesar (Formerly Biomedical Technologies, USA). Forskolin (adenylyl cyclase activator) and rolipram (PDE IV-inhibitor) were used as a cAMP- increasing drug combination and were purchased from Sigma-Aldrich (MO, USA).

### Human tissue samples

Carotid and Femoral artery samples from an autopsy of a CVD patient were generously provided by the Queens Medical Center (HI, USA). The artery samples were collected after obtaining consent from a family member and reviewed by the Research and Institutional Review Committee of the Queens Medical Center. The tissues were frozen immediately and kept at −80 °C in the hospital before being transported on dry ice to the University of Hawaii. A portion of each tissue sample was either embedded and frozen in OCT (Sakura Finetek, USA), cut into 10uM sections and analyzed by polarized light microscopy, or prepared as described above for TEM analysis.

### Cell culture

Primary human aortic endothelial cells (HAoEC, Cat#: C-12272), primary human umbilical vein endothelial cells (HUVEC, Cat#: C-12253), primary human skin fibroblasts (HSF, Cat#: C-12352) as well as primary human aortic smooth muscle cells (VSMC, Cat#: C-12532) (all PromoCell, Germany) were cultured according to manufacturer’s recommendation using the corresponding media as well as the split kit (all, PromoCell, Germany). Cells were used up to passage 8. Human monocyte-derived macrophages (HMDM) were isolated from whole blood from consenting healthy volunteers by Histopaque (Sigma-Aldrich) density gradient centrifugation. Informed consent was obtained from all donors and IRB approval was received under protocol CHS# 19345 prior to experiments involving HMDM. Cells from the buffy coat were plated with hematopoietic X-VIVO 15 medium (purchased from Lonza, Switzerland) supplemented with 20% heat inactivated human serum for 1 hour before non-adherent cells were washed away. Adherent cells were maintained for 5 days on glass slide chambers before being used in experiments.

### In vitro HAoEC treatment

HAoEC were cultured for 3 days after splitting. At day 4, cells were treated with 50 μg/ml LDL (low dose), 250 μg/ml LDL (high dose), 50 μg/ml AcLDL or OxLDL or 100 μg/ml DiI-LDL. After 3, or 5 days of treatment, cells and their according controls were washed with PBS, fixed and prepared for subsequent PL and confocal microscopy. For PL as well as confocal microscopy, cells were fixed for 10 min at room temperature using 4% PFA. DiI-LDL-treated samples were counterstained with DAPI, mounted with DAKO fluorescence mounting media and analyzed using confocal microscopy (Olympus FV-1000). For SEM and TEM analysis, cells were fixed using 4% glutaraldehyde and 0.1 M calcium chloride in 0.1 M sodium cacodylate buffer (pH 7.2) for 24 h.

HAoEC, HUVEC, HSF and HMDM were treated with 50 μg/ml LDL (low dose), 250 μg/ml LDL (high dose), 50 μg/ml AcLDL or OxLDL or 100 μg/ml DiI-LDL. After 5 days of treatment, cells were fixed using 4% PFA and subjected to PL microscopy. A second set of treated cells was fixed and permeabilized using 0.1% TritonX-100 in PBS for 5 min at RT. The actin cytoskeleton as well as the nuclei of these cells was labeled using Phalloidin-Alexa488 and DAPI, respectively and mounted using DAKO fluorescence mounting media (DAKO). Fluorescently labeled cells were analyzed and photographed using an Axiovert (Zeiss, Germany) microscope.

### Preparation of cell culture dishes with CC and gelatin

HAoEC used for experiments were cultured on gelatin or gelatin+CC coated surfaces. Cell culture dish surfaces were covered completely using 0.5 mg/ml gelatin in PBS or 0.5 mg/ml gelatin in PBS plus 1 mg/ml CC and incubated at 37 °C for 1 h. Afterwards surfaces were gently rinsed three times using PBS. Coating of surfaces was prepared fresh for each experiment.

### Endothelial barrier integrity measurement using ECIS

An electric cell substrate impedance-sensing set-up (ECISZΘ, Applied BioPhysics Inc, Troy, NY, USA) was used to measure and analyze the transendothelial electrical resistance (TER) as a measure of HAoEC barrier integrity. Electrode arrays were coated as described above and equilibrated for 1 h in the incubator. HAoEC were seeded into these equilibrated ECIS arrays and the measurement started immediately. By measuring the capacity and the impedance/TER we were able to detect the process of cell adherence and endothelial barrier formation over time (24 h). Afterwards, cells were treated with forskolin/rolipram (5 μM/10 μM) and changes in endothelial barrier function observed by ongoing measurement of TER at 4000 Hz. In case of wounding/regeneration experiments a current of 30,000 Hz for 30 s per well was applied. Recovery of the wounded area was observed by ongoing measurement. Obtained data were analyzed using ECIS software and Microsoft Excel.

### Trans-endothelial migration in a transwell filter setup

Transmigration experiments in vitro were performed using human monocyte THP-1 (ATCC number TIB-202) and T-cell Jurkat (ATCC number TIB-152) cell lines, which were purchased directly from ATCC (which included certifications of analysis but were not further tested for mycoplasma). HAoEC were cultured on gelatin or gelatin + CC coated 5 µm pore size transwell filters for 5 days. At day 5, 2.5 × 10^5^ THP-1 or Jurkat cells per cm^2^ along with fresh media were added to the HAoECs and were allowed to transmigrate through the endothelium and the coated filters into the lower compartment supplemented with 100 ng/ml SDF-1 or 20 ng/ml MCP-1 for 24 h. F/R treatment consisted of 5 μM/10 μM forskolin/rolipram for 1 h before starting the transmigration experiment. Transmigrated live cells were labeled with Calcein and counted using a BD FACS Calibur.

### Detection of RhoGTPase activity and intracellular cAMP level

HAoEC were cultured on gelatin or gelatin + CC coated 6-well plates. At day 5 cells were F/R treated, harvested and analyzed for RhoA, Rac1, Cdc42 and Ras activity as well as for their cAMP levels according to manufactures protocol. GLISAs were purchased from Cytoskleton Inc and cAMP ELISA was purchased from EnzoLifeSciences.

### Flow cytometry analysis

HAoEC were cultured for 5 div on gelatin or gelatin+CC. At day 5 HAoEC were trypsinized and stained for ICAM-1 (a-ICAM-1-FITC), VCAM-1 (a-CD106-APC) and E-selectin (a-CD62E-PE) for 20 min on ice in the dark. The expression of these intercellular adhesion molecules was analyzed by flow cytometry using the FACS Calibur.

Secondly, *Ldlr* ^*−/−*^ mice were put on HFD for 0–3 weeks, the aorta isolated and thoroughly cleaned, digested using Liberase^TM^ (Roche), as described previously^50^ and E-selectin expression was quantified using flow cytometry. Data were analyzed using FlowJo software according to the flow chart in Supplementary Fig. 9b. To verify the functionality of the antibody used against mouse E-selectin, we utilized the MS1 mouse endothelial cell line (ATCC® CRL-2279™) stimulated with various concentrations of TNFα and observed a concentration dependent increase of E-selectin positively stained cells (Supplementary Fig. 9a). Digested aortas were also analyzed for the presence of CD11b and CD11c positive cells. Further information on the antibodies used can be found in Supplementary Table 1.

### Analysis of lipids in HAoEC and aortic root sections

For visualization of esterified lipids in the incubated cells or in the aortic root sections, Oil Red O staining was used as described previously^51^. For quantification purposes, treated HAoEC were stained using Bodipy-Alexa488 (Invitrogen, USA) and analyzed using flow cytometry (FACS Calibur, BD). To measure free cholesterol content, HAoEC were stained using filipin (Sigma) and analyzed by flow cytometry (Beckman-Coulter Altra).

### Transmission electron microscopy (TEM)

For TEM, specimens were fixed with 4% glutaraldehyde and 1 M calcium chloride in 0.1 M sodium cacodylate buffer, pH 7.2 for 24 h and washed in 0.1 M cacodylate for 3 × 10 min, followed by post-fixation with 1% OsO4 in 0.1 M cacodylate buffer for 1 h. Cells and tissue were dehydrated in a graded ethanol series (30%, 50%, 70%, 85%, 95%, 100%), substituted with propylene oxide, and embedded in LX112 epoxy resin. Ultrathin (60-80 nm) sections were obtained with a diamond knife on an RMC Powertome ultramicrotome, and double stained with uranyl acetate and lead citrate. Grids were viewed and photographed on a Hitachi HT7700 transmission electron microscope operating at 100 kV with an AMT XR41 4 megapixel camera (Advanced Microscopy Techniques, Corp.).

HAoEC subjected to TEM were grown and fixed in tissue culture treated 35mm dishes, fixed and prepared as described above, with the exemption that no propylene oxide was used.

### Scanning electron microscopy

For SEM, specimens were fixed and processed exactly as with TEM through ethanol dehydration, at which point the samples were critical point dried, mounted on aluminum stubs, sputter coated with gold/palladium, and viewed with a Hitachi S-4800 field emission scanning electron microscope at 5 kV. For energy-dispersive X-ray spectroscopy (EDS), spectra were acquired operating the SEM at 20 kV field emission and using an Oxford INCA PentaFET-x3 Si (li) EDS detector.

HAoEC that were subjected to SEM were grown and treated on 12mm glass coverslips and prepared as described above. Mouse aortas were cracked open using cactus spines after mounting to the aluminum stubs to allow SEM analysis of a ‘cross section’ of the vessel wall.

### Energy-dispersive X-ray spectroscopy (SEM/EDS)

For energy-dispersive X-ray spectroscopy (EDS) samples in the SEM were used, spectra were acquired operating the SEM at 20 kV field emission and using an Oxford INCA PentaFET-x3 Si (li) EDS detector.

### Atherosclerosis model and analysis

All animal protocols were approved by the University of Hawaii Institutional Animal Care and Use Committee. For initial analysis of aortic changes after hyperlipidemia, male *Ldlr* ^*−/−*^ mice on C57BL/6 background 8-10 weeks of age were put on a high fat diet (HFD) containing 15.8% (wt/wt) fat and 1.25% cholesterol (94059; Harlan Teklad) for 1 or 2 weeks. In a second set of animal experiments, 4 groups of 8-week old male *Ldlr* ^*−/−*^ mice were kept on HFD, half for 1 week and half for 2 weeks. The first 2 groups on HFD for 1 week were intraperitoneally (IP) injected every 48 h with: (a) PBS or (b) a combination of 2 mg/kg forskolin and 0.5 mg/kg rolipram. The second 2 groups were put on HFD for 1 week, then during the second week of HFD were intraperitoneally (IP) injected every 48 h with: (a) PBS or (b) a combination of 2 mg/kg forskolin and 0.5 mg/kg rolipram. In a third set of experiments, 6 week old male *ApoE* ^*−/−*^ mice were IP injected with (a) PBS + untargeted liposomes, (b) 1 μg/kg TNFα + untargeted liposomes, (c) untargeted liposomes and fed a HFD, (d) targeted liposomes, (e) 1 μg/kg TNFα + targeted liposomes and (f) untargeted liposomes and fed a HFD every 48 h for 1 week. In a fourth set of experiments 6 week old male *ApoE* ^*−/−*^ mice were IP injected with (a) PBS and (b) 2 mg/kg forskolin and 0.5 mg/kg rolipram every 48 h while tail-vein injected with (c) 200 μl PBS-targeted liposomes or (d) 200 μl 5 μM forskolin/10 μM rolipram targeted liposomes once a week for 6 weeks while on HFD.

Upon killing, the mouse hearts and aortas were collected and processed for further analysis. Two different portions of the aortic arch were used for SEM and TEM analyses. These samples were fixed immediately as described above. The aortic sinus was cut into 10μm sections and analyzed using polarized light microscopy, Oil Red O staining and immunohistochemistry.

### Immunofluorescence of aortic root sections and HAoEC

The immunofluorescent visualization of macrophages and smooth muscle cells in the aortic root sections were done with a Rat-α-MOMA-2 (AbCAM) as well as Rabbit-α-SM22α (ProteinTech) primary antibodies and Goat-α-Rat Cy2- and Goat-α-Rabbit Cy3-labeled secondary antibodies, respectively, as described previously^51^.

Immunofluorescent visualization of VE-cadherin and claudin5 in HAoEC was performed after fixation of HAoEC using 4% PFA in PBS (pH 7.4) for 10 min at RT, permeabilization using 0.1% TritonX-100in PBS for 5 min at RT and blocking using 2% BSA/10% normal goat serum in PBS for 30 min at RT. Primary antibodies were added for overnight incubation at 4 °C. Cy3-labeled secondary antibodies were used, F-actin and nuclei were stained with Phalloidin-Alexa488 and DAPI, respectively. Samples were mounted in DAKO mounting media (DAKO) and visualized using a Zeiss Axiovert microscope. Further information on antibodies used can be found in Supplementary Table 1.

### sLe^x^-liposome treatment of HAoEC

sLe^x^-liposomes were created by Dr. Noboru Yamazaki (details under US patent application #20090169610) and designed to bind E-selectin on the surface of target cells. We obtained both targeted and untargeted liposomes, with or without sLe^x^ respectively, from Dr. Yamazaki (now at Innomedica). Liposomes containing DiI were used to characterize effective binding to target cells using fluorescence, while liposomes containing F/R were used for the atherosclerosis studies described. HAoEC were grown to confluence on 12-well plates or 8-chambered well glass slides and treated with DiI-filled untargeted or targeted liposomes, with or without TNFα (10ng/ml) stimulation. For confirmation of F/R delivery via targeted liposomes, HAoEC were grown in 6-well plates and treated as indicated and lysates were harvested as described above. cAMP concentration was measured using a cAMP assay (Enzo Life Sciences, USA) according to the manufacturer’s protocol as described previously^25, 52^.

### Preparation of sLe^x^-labeled liposomes

The liposomes were prepared using the improved cholate dialysis method^53^. Dipalmitoylphosphatidylcholine (16.8 mg), cholesterol (10.1 mg), diacetylphosphate (1.8 mg), ganglioside (14.6 mg), dipalmitoylphosphatidylethanolamine (2.3 mg), and sodium cholate (46.9 mg) were mixed and dissolved in 3 ml of chloroform/methanol (1:1, v/v) solution. The solvent was evaporated using a rotating evaporator at 30 °C and the lipid film was obtained after drying under vacuum. This lipid film was dissolved in 3 ml of tris(hydroxymethyl) methylaminopropanesulfonic acid buffer (TAPS, pH 8.4), and the micelle suspension was then obtained after sonicating. The labeling method for binding Cy5.5 to human serum albumin (HSA) is as follows. Twenty mg of HSA and 2 mg of Cy5.5-NHS ester (GE Healthcare) were dissolved in 3 ml of TAPS (pH 8.4) and stirred at 37 °C for 3 h. To remove residual Cy5.5-NHS ester, the solution was ultrafiltrated with TAPS (pH 8.4) using an 8010 ultrafiltration cell (Amicon) fitted with an PM10 membrane (Amicon). Three milliliter of HSA with Cy5.5 solution was mixed with the micelle suspension above, and this was ultrafiltered with TAPS (pH 8.4) by PM10 membrane to remove residual HSA with Cy5.5, then 10 ml of liposome solution was obtained. In addition, the solution was ultrafiltered with sodium hydrogen carbonate buffer (CBS, pH 8.5) by XM300 membrane (Amicon) to exchange buffer. Ten milligram of bis(sulfosuccinimidyl)suberate (BS_3_, Pierce), a crosslinking agent, was added to 10 ml of liposome solution, and stirred at 20–25 °C for 2 h, then further stirred overnight at 4 °C. BS_3_ was combined to the liposome surface. Then, 40 mg of tris(hydroxymethyl) aminomethane (Tris) was added, and stirred at 20–25 °C for 2 h, then further stirred overnight at 4 °C to bind Tris to BS_3_. This was ultrafiltered with TAPS (pH 8.4) by XM300 membrane to remove residual Tris. To bind HSA to the liposome surface, the coupling method was used^53^. To oxidize the liposome surface, 10.8 mg of sodium periodate was added to 10 ml of liposome solution and stirred at 4 °C overnight. To remove residual sodium periodate, it was ultrafiltered with phosphate saline buffer (PBS, pH 8.0) by XM300 membrane. Twenty milligram of HSA was added to it and stirred at 20–25 °C for 2 h. Then 3.13 mg of sodium cyanoborohydrate was added, and stirred at 20–25 °C for 2 h and further overnight at 4 °C. To remove residual sodium cyanoborohydrate, the solution was ultrafiltered with CBS (pH 8.5) by XM300 membrane. Sugar chains were combined on the liposome surface through 3,3-dithiobis(sulfosuccinimidylpropionate) (DTSSP, Pierce). DTSSP was used as a cross-linking reagent. Ten milligram of DTSSP was added to 10 ml of liposome solution and stirred at 20–25 °C for 2 h, and further overnight at 4 °C. To remove residual DTSSP, the solution was ultrafiltrated with CBS (pH 8.5) by XM300 membrane. The amination of the reducing group terminal of sugar chain was done by the glycosyl amination reaction. Two milligram of SLX (Calbiochem) was dissolved in 0.5 ml of distilled water. A total of 0.25 g of NH_4_HCO_3_ was added and stirred at 37 °C for 3 days. Aminated SLX was added to reach a final concentration of 100 μg/ml and stirred at 25 °C for 2 h. Tris was then added to reach a final concentration of 132 mg/ml, and stirred overnight at 4 °C for repeated hydrophilization of the liposome surface. To remove residual SLX and Tris, the solution was ultrafiltered with *n*-(2-hydroxyethyl)piperazine-*n*′-(2-ethanesulfonic acid) (Hepes, pH 7.2) by XM300 membrane. The preparation of liposome without sugar chains was similar to the SLX-Lipo-Cy5.5 case except for the process for binding sugar chains.

### Statistics

For in vitro experiments, at least three biological replicates were analysed, with 2 or 3 technical replicates run for each assay as indicated. For the in vivo mouse studies comparing 2 groups with 8 mice per group and a resulting degree of freedom of 14, a student t-distribution of 2.3 is used as the limitation for statistical significance with alpha = 0.05 (two-tailed test). With a power of 80% and standard deviation within groups < 20% from the mean, which is typical for the plaque and other analyses, a minimum of 8 mice per group was set for detecting significant differences of 25% between groups. Mice were randomly assigned to treatment groups with littermates divided evenly between groups. Blinding was not performed for experiments. Comparison of means between groups with normal sample distribution was performed with Prism software (GraphPad) using the Student’s unpaired, two-tailed t-test. In the case of 3 or more groups, one way ANOVA was performed. Any outlier values were determined by statistical test using Prism and excluded from data sets. Data are presented as mean±the standard error of the mean (SEM). Statistical significance was accepted at the level of *p* < 0.05.

### Data availability

The data that support the findings of this study are available from the corresponding author upon request.

## Electronic supplementary material

Supplementary Information
