# Supplementary material for: Hyperlipidemia-induced cholesterol crystal production by endothelial cells promotes atherogenesis
Source: Nat Commun. 2017 Oct 24;8:1129. doi: 10.1038/s41467-017-01186-z (PMC5654750; doi:10.1038/s41467-017-01186-z)
Supplement: Supplementary file 1 — Supplementary Information [file 41467_2017_1186_MOESM1_ESM.pdf]

Supplementary Information

Supplementary Figures

Supplementary Figure 1

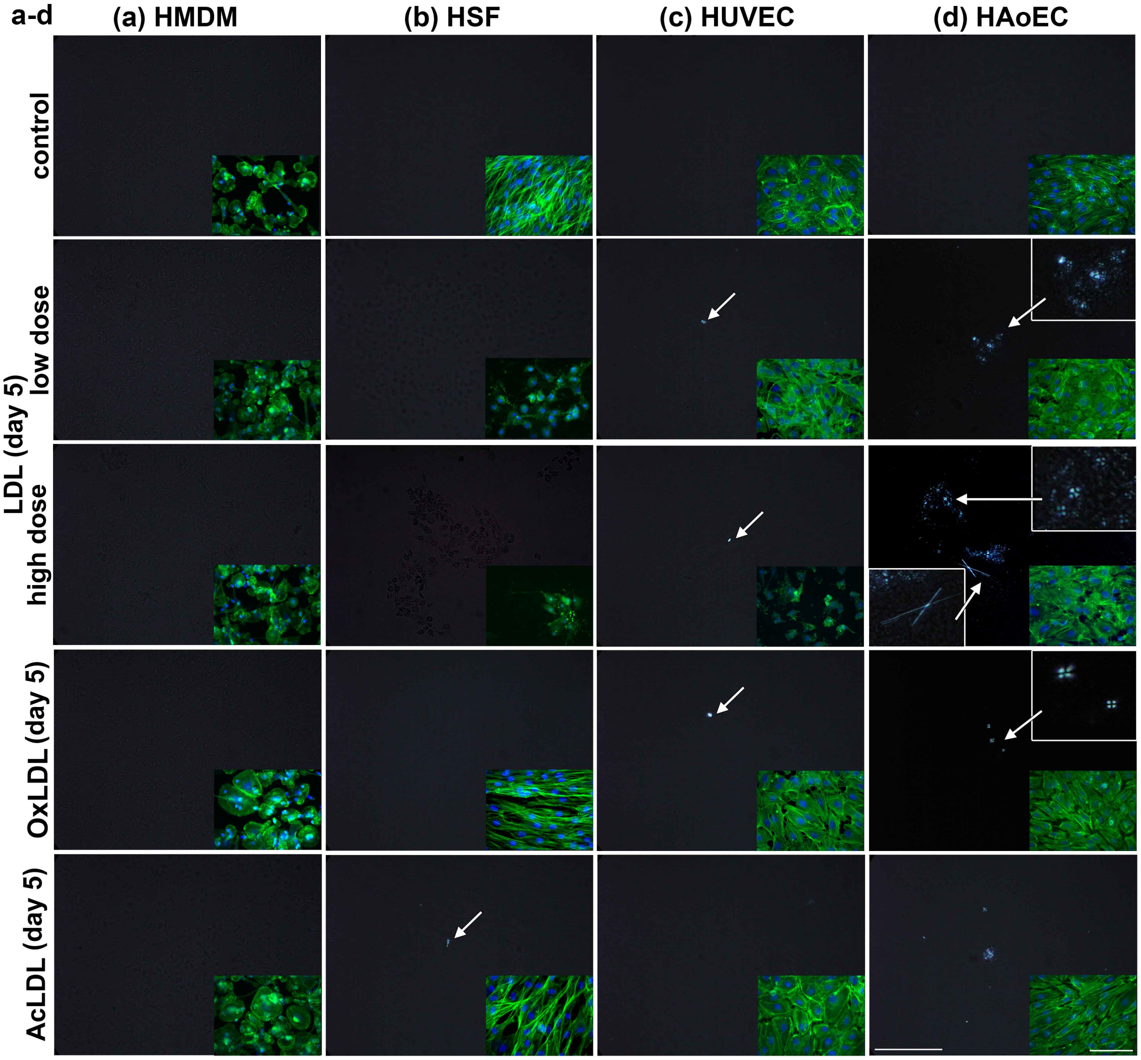

Supplementary Figure 1: Cholesterol crystals produced exclusively in EC of aortic origin. (A-D) Four different primary human cell types were treated with high and low dose of LDL, OxLDL and AcLDL for 5 days before analyzed for the presence of CC using PL. **(a)** Human monocyte-derived macrophages (HMDM) from whole blood as well as **(b)** human skin fibroblasts (HSF) failed to form any CC with any of the treatments. To determine if this CC formation occurs exclusively in arterial ECs, human umbilical vein endothelial cells (HUVECs) were also tested. **(c)** HUVECs displayed almost no CC formation compared to HAoECs. **(d)** In HAoECs, even low dose LDL treatment displayed spherulite formation within 5 days, while high dose LDL treatment led to additional needle shaped CC formation. Treatment with AcLDL did

not induce EC-derived CC formation while oxLDL induced spherulite formation. (representative images of n=3 are shown, scale bars = 50μm)

<u>**Supplementary Information**</u>

Supplemental Figure 2

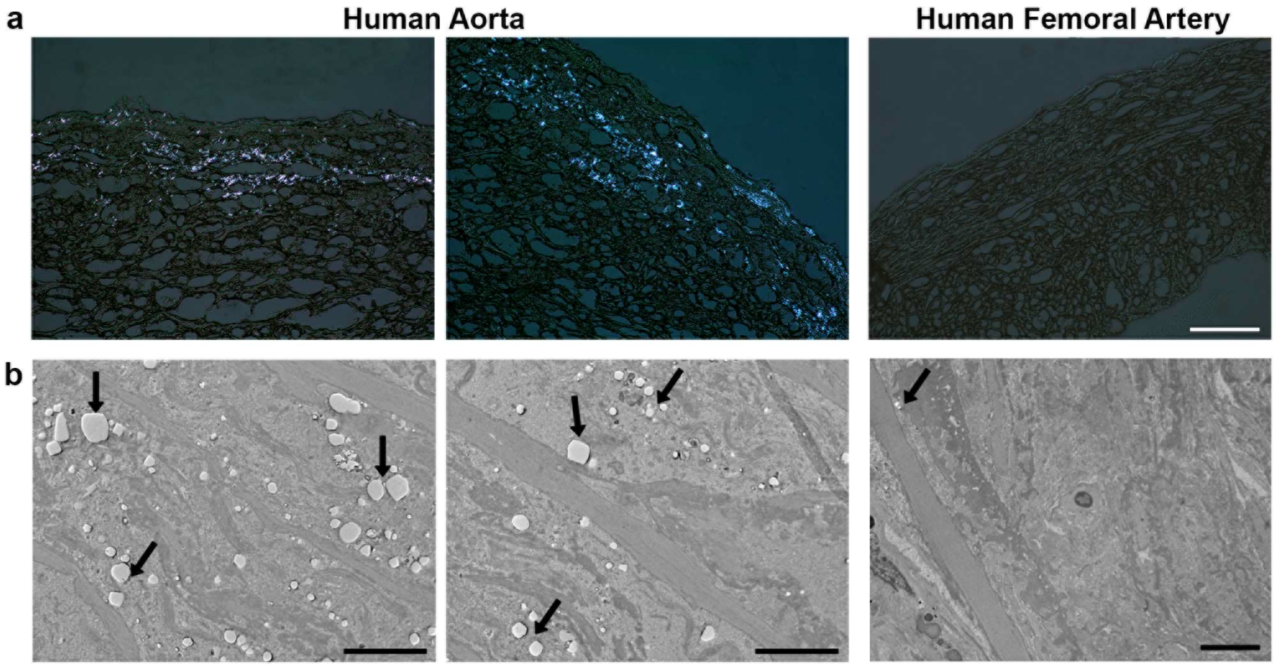

Supplementary Figure 2: Detection of cholesterol crystals (CC) in human atherosclerotic tissue. Atherosclerotic human aorta as well as control femoral artery were subjected to polarized light (PL) **(a)** and transmission electron microscopy (TEM) **(b)**. CC deposition within the aorta is clearly visible under polarized light while the control femoral artery showed no CC deposition. CC in the aorta can be seen as “clefts”. These clefts are being detected in the same patient’s control femoral artery. (scale bar PL = 500μm, TEM = 500nm)

Supplementary Information

Supplementary Figure 3

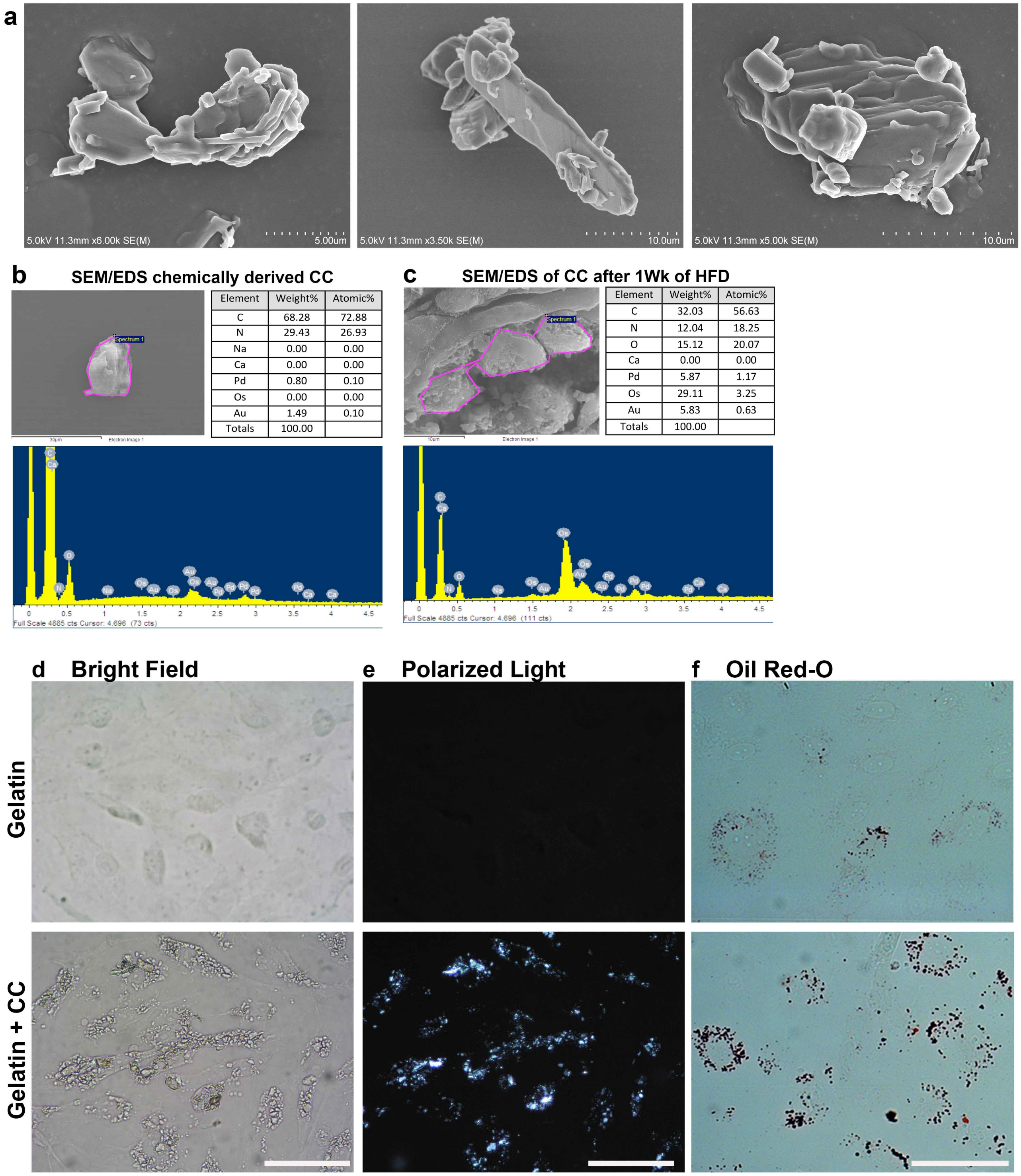

Supplementary Figure 3: Characterization of synthetic cholesterol crystals. (a) SEM analysis of the CC displays plate-shaped agglomerates of various sizes. To simulate *in vivo* conditions, CC were embedded into a gelatin coating **(b-c)** SEM/EDS analysis was performed on chemically derived CC **(b)**

and CC found underneath the aortic endothelium in *Ldlr*$^{-/-}$ mice after 1 week of HFD **(c**). Both cholesterol crystal types show a remarkably similar makeup, with no Calcium present. Osmium cannot be detected in chemically derived CC due to those crystals not being osmium fixed for sample preparation. Gold and palladium are present due to the Au/Pd coating necessary for SEM imaging. (25 chemically derived CC were examined as well as 10 areas each on three 1 week HFD aortas) **(d)** Bright field images of HAoEC grown on gelatin or gelatin+CC is shown, while PL analysis **(e)** reveals CC are equally distributed in the gelatin+CC coating with gelatin coating alone displaying none. Cultivation of HAoECs on gelatin or gelatin+CC coating for 5 days shows subendothelial uptake of CC exclusively in cells cultivated on gelatin+CC coating. Oil Red O staining **(f)** for esterified lipids revealed that HAoECs cultivated on gelatin+CC coating display large amounts of lipid uptake while control cells on gelatin alone display only a few positively stained areas likely from lipids in the cell culture medium. (Representative images of n=3 are shown, scale bar d-f= 50μm)

Supplementary Information

Supplementary Figure 4

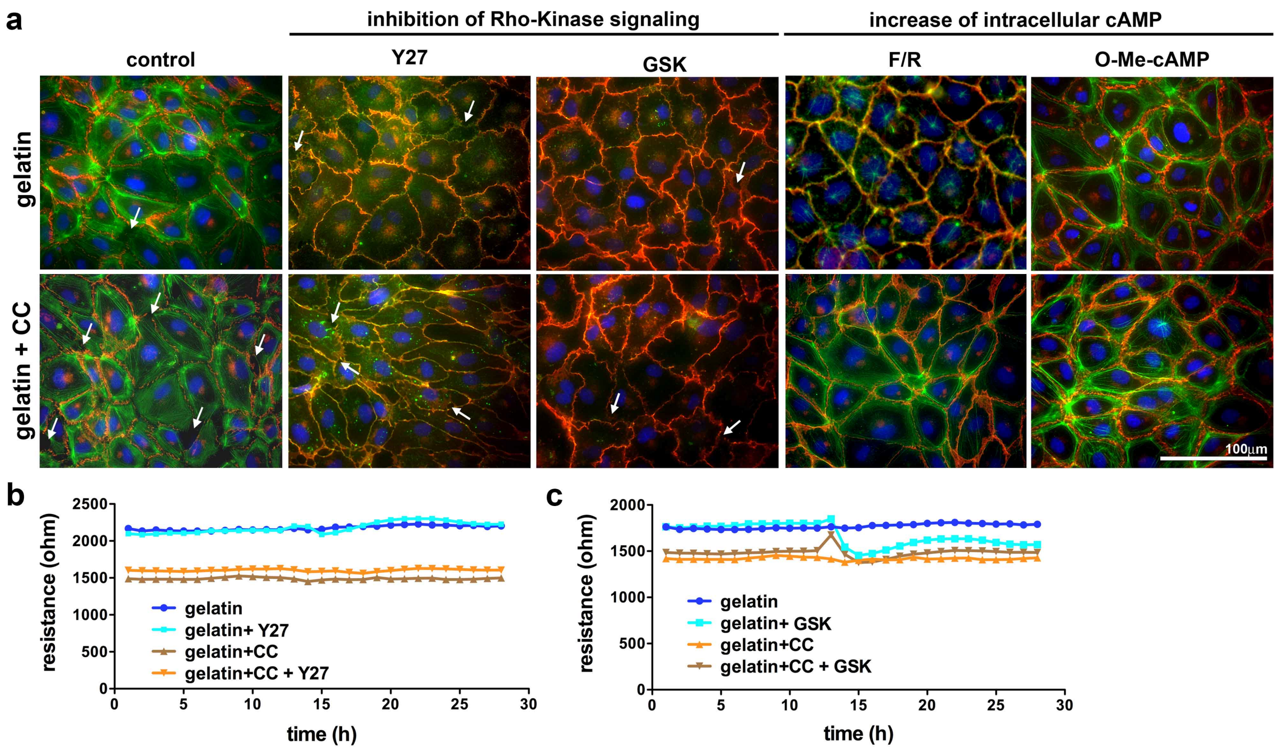

Supplementary Figure 4: Impact of Rho-Kinase signaling on subendothelial CC-induced endothelial dysfunction. HAoEC were grown to confluence for 7 days on gelatin and gelatin+CC coated surfaces. After reaching confluence cells were treated with Rho-Kinase inhibitors Y27632 and GSK429286 as well as with cAMP-enhancing agents F/R and O-Me-cAMP for 1h. **(a)** Subsequently adherence junction VE-cadherin (red) was stained and actin cytoskeleton labeled using Phalloidin (green, nuclei = blue/DAPI). In parallel a second set of cells was monitored using ECIS and treated as described above **(b)**. Immunofluorescence and barrier integrity measurement using ECIS show that inhibition of Rho-Kinase is not sufficient to restore endothelial barrier integrity nor intercellular gap formation (white arrows), while increase of cAMP restores barrier integrity by tightening adherence junctions with no intercellular gaps detectable. (Representative images or ECIS curve of n = 3 shown, scale bar = 100µm)

Supplementary Information

Supplemental Figure 5

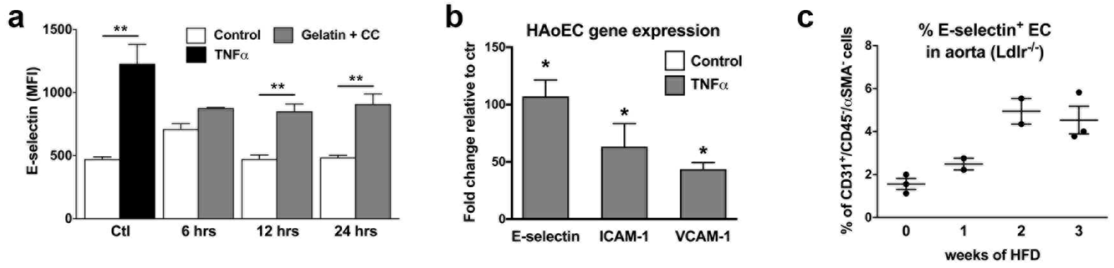

Supplementary Figure 5: Endothelial E-selectin expression in HAoEC and mouse aortas. (a) Flow cytometry for membrane-expressed E-selectin on HAoEC after seeding and culturing on gelatin+CC for 6, 12, and 24 hours. Cells grown on gelatin alone and treated with 10ng/ml TNFα served as positive control. (n=4, error bars represent SEM, *p<0.05) **(b)** Gene expression of ICAM-1, VCAM-1, and E-selectin in HAoEC was determined after 6h 10ng/ml TNFα treatment, with E-selectin being the highest expressed gene (n=4, error bars represent SEM, *p<0.05). **(c)** *Ldlr$^{-/-}$* mice were put on HFD for 0-3 weeks. Upon sacrifice aortas were excised, thoroughly cleaned and digested using Liberase^{TM}. Cell suspension was analyzed for E-selectin expression on CD31-positive cells, showing an increase from 1 week of HFD on. (error bars represent SEM, n=2-3 per time point)

Supplementary Information

Supplementary Figure 6

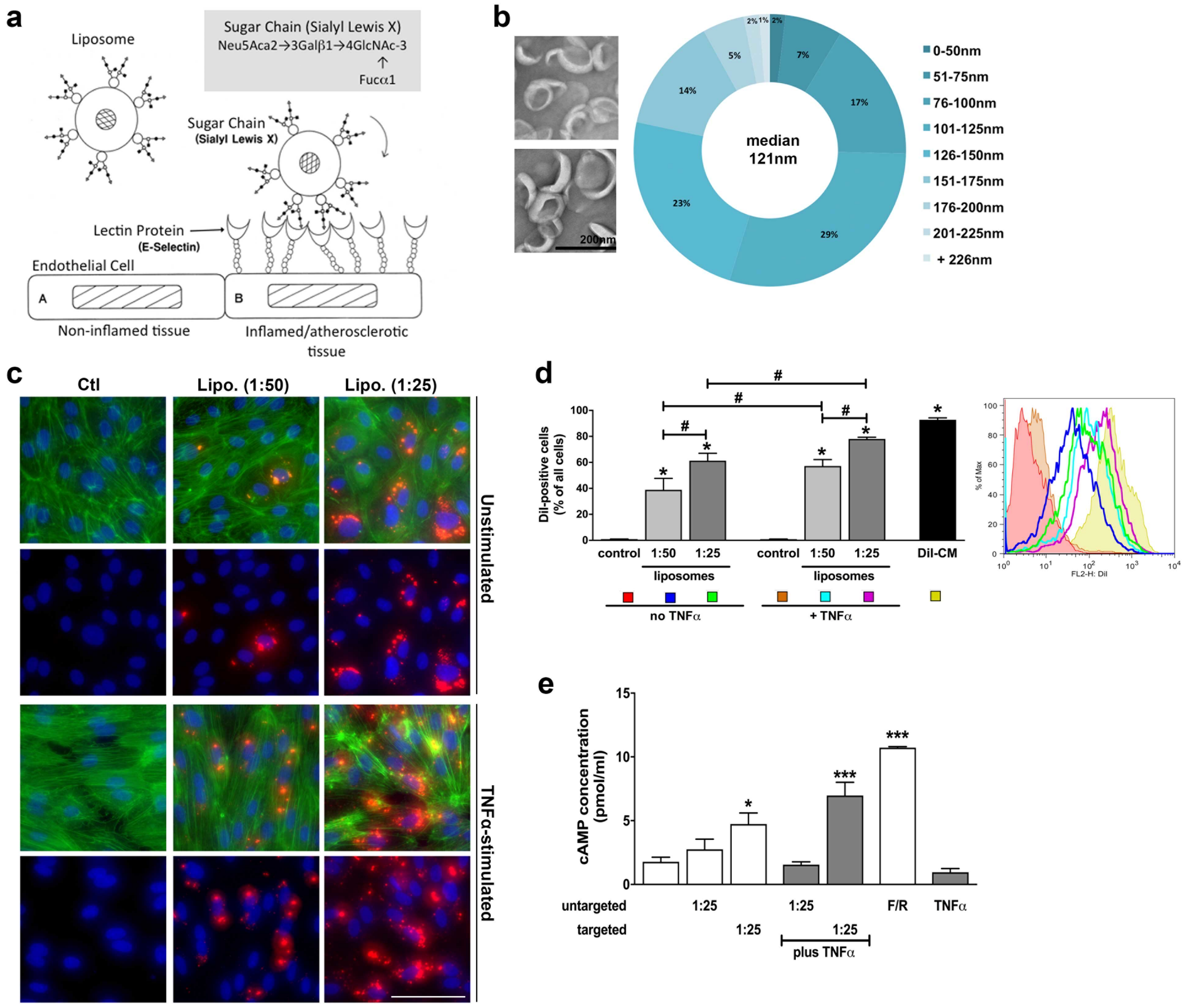

Supplementary Figure 6: sLe^{x} liposomes description and characterization of binding to inflamed endothelial cells *in vitro*. (a) Schematic diagram showing the mechanism behind the targeted delivery of sLe^{x} liposomes to inflamed EC expressing E-Selectin. **(b)** TEM analysis of the liposomes bound to EC *in vitro* with the size range observed primarily between 76nm to 150nm, with a median size of 121nm. **(c)** EC uptake of DiI-filled liposomes *in vitro* was tested with or without TNFα for 24 hrs. Liposome dilutions of 1:50 and 1:25 were tested. Untreated and DiI-CM-treated cells used as negative and positive controls respectively, with red fluorescence indicating liposome uptake or DiI-CM, nuclei stained with DAPI in blue, and the actin cytoskeleton labeled with Phalloidin in green. (Scale bar = 50μm) **(d)** Flow cytometry analysis and quantification of DiI fluorescence following treatment of HAoEC with DiI-filled liposomes or

Supplementary Information

DiI-CM. **(e)** cAMP concentration measured in HAoEC after treatment with untargeted or targeted liposomes containing F/R. Treatment with free F/R or 10ng/ml TNFα were used as positive and negative controls respectively. (n=4, error bars represent SEM, *$p<0.05$, **$p<0.01$, ***$p<0.001$)

Supplementary Information

Supplementary Figure 7

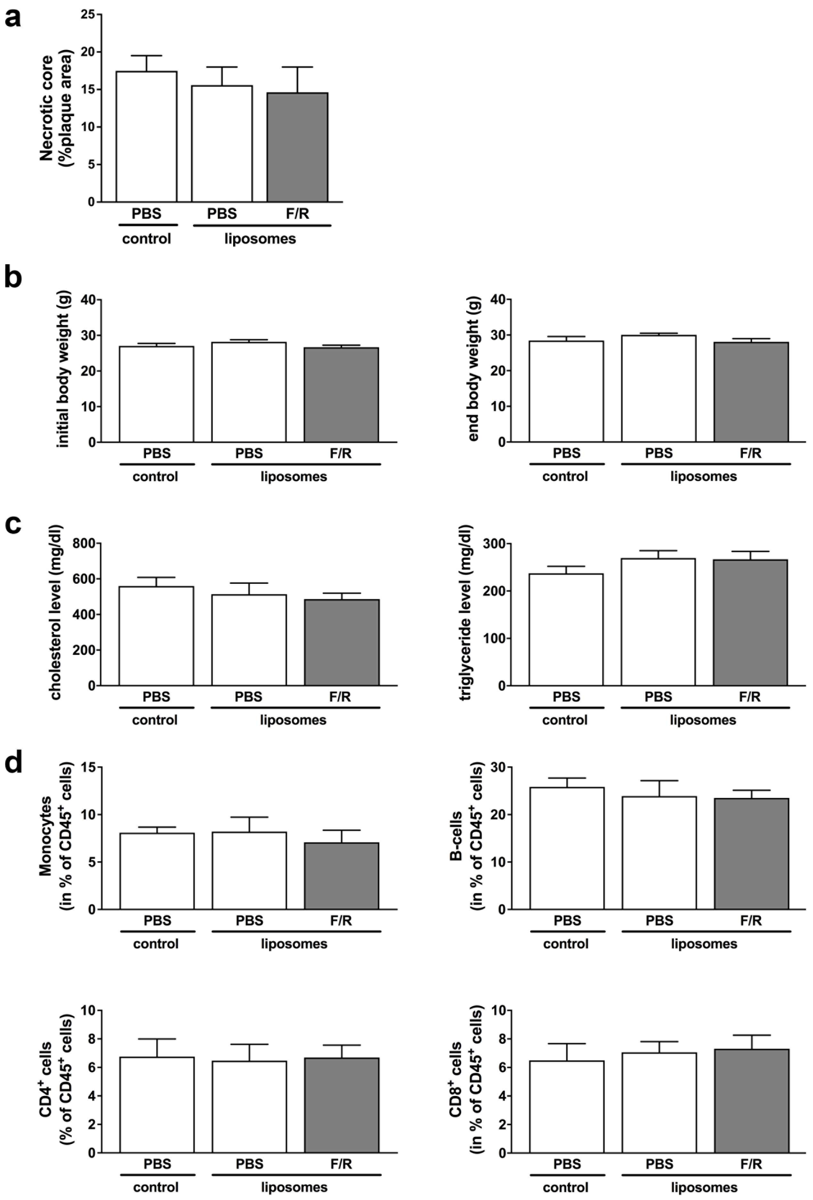

Supplementary Figure 7: Physiologic parameters of mice after the 6-week HFD atherosclerosis study. (**a**) Necrotic core size presented as (% of plaque). (**b**) There was no significant difference between PBS control and F/R treated mice in weight (**b**). Serum was collected from whole blood and analyzed for cholesterol and triglyceride levels **(c)**, which were found to have no difference between groups. Analysis of peripheral monocytes, B-cells, as well as $CD4^+$ and $CD8^+$ T-cells after 6 weeks of HFD also showed no significant differences **(d)**. (n=8, error bars represent SEM)

Supplementary Information

Supplemental Figure 8

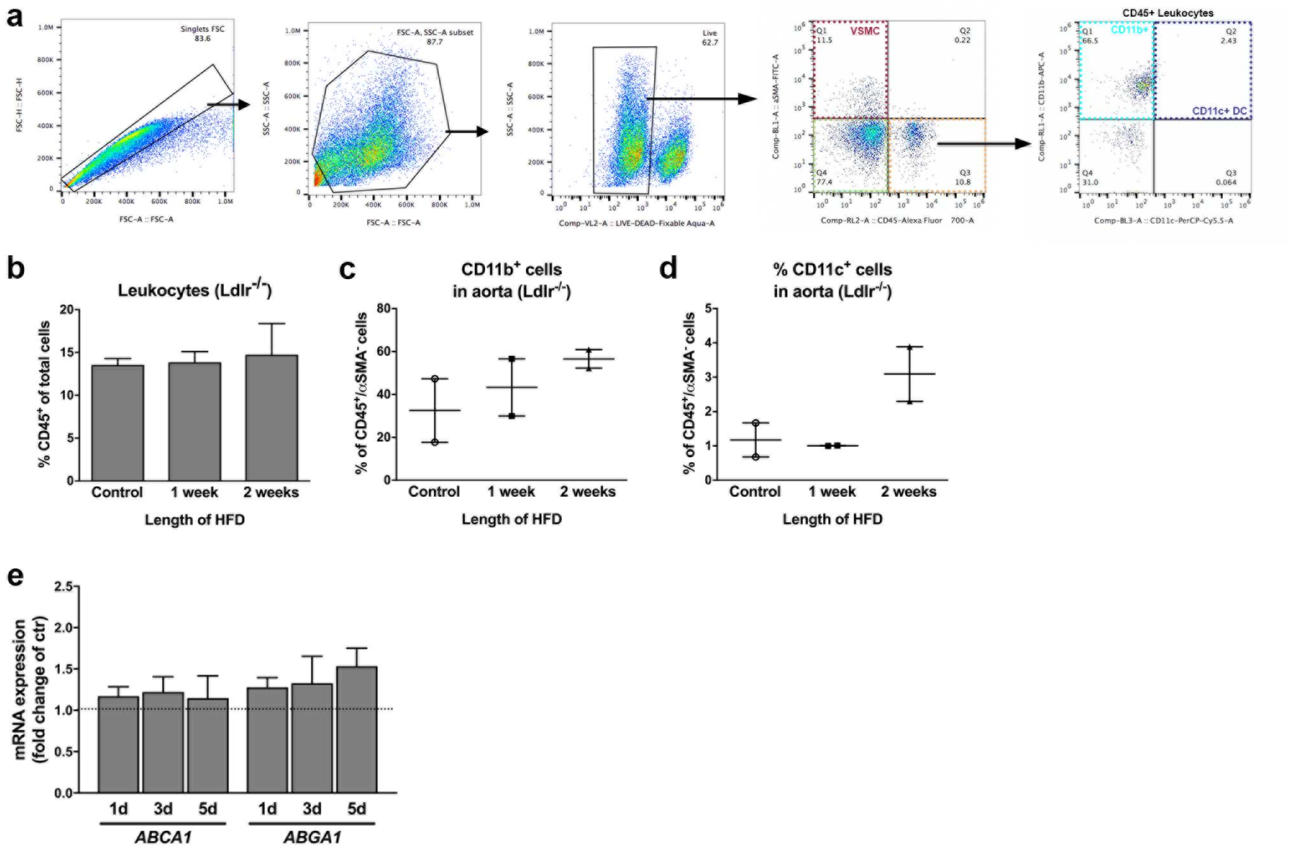

Supplementary Figure 8: Additional data incorporated in discussion. (**a**) Flow diagram used to analyze the presence of myeloid cells within *Ldlr*$^{-/-}$ mouse aortas after Liberase^{TM} digest. (**b**) CD45+ leukocyte population in *Ldlr*$^{-/-}$ mouse aortas after 0-2 weeks of HFD. (n=3, error bars represent SEM) (**c-d**) Subpopulations of CD11b^{+} and CD11c^{+} populations in *Ldlr*$^{-/-}$ mouse aortas after 0-2 weeks of HFD. (n=2, error bars represent SEM). (**e**) mRNA expression of efflux transporters *ABCA1* and *ABCG1* on 1, 3 and 5 days LDL treated HAoECs.

Supplementary Information

Supplemental Figure 9

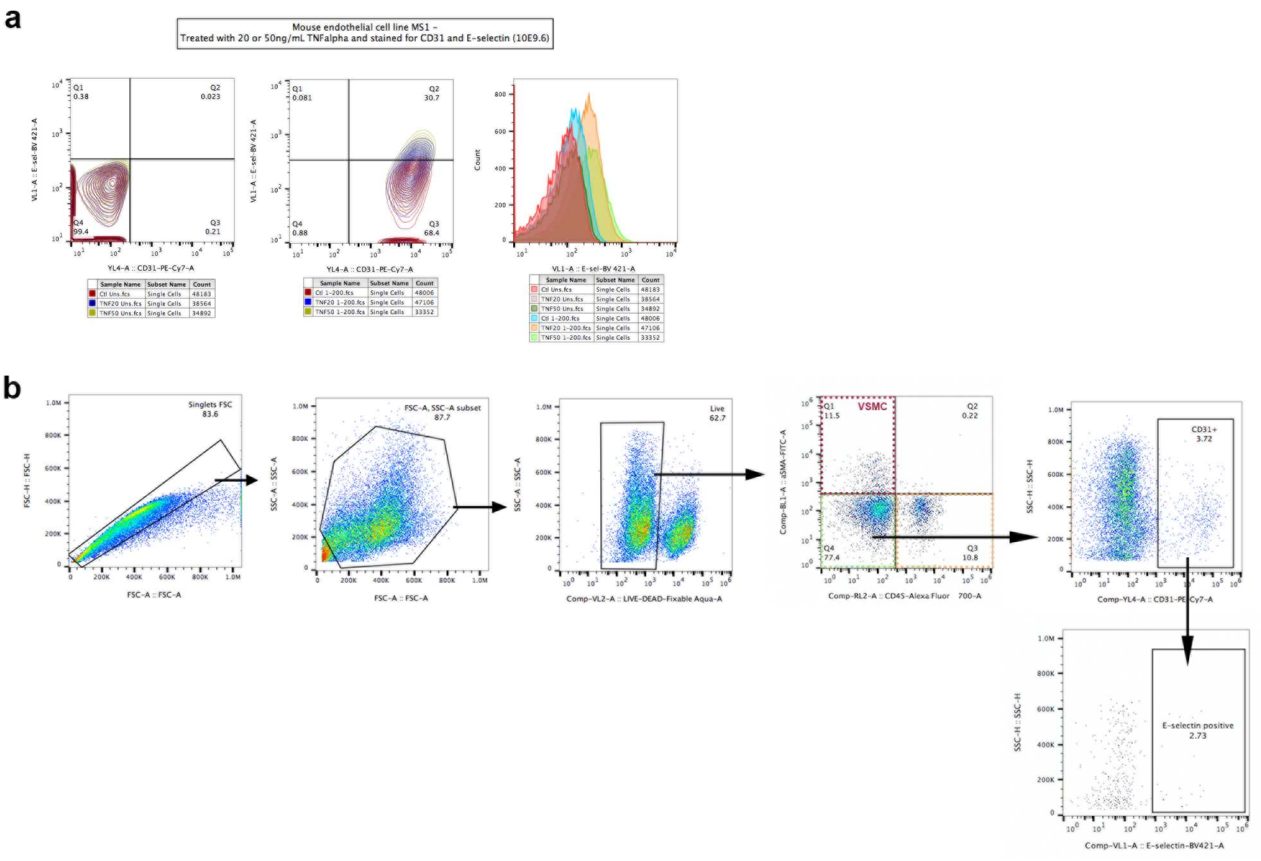

Supplementary Figure 9: Additional data incorporated in Material and Methods. (**a**) Verification of anti-mouse E-selectin antibody. Mouse endothelial cell line MS1 was treated with different doses of TNFα, stained for E-selectin and CD31, and subsequently the MFI for E-selectin determined. Almost all MS1 cells were CD31 positive with variating levels of E-selectin, confirming that the E-selectin antibody (10E9.6 clone) is functional and can be used for experiments. (**b**) Flow chart to determine E-selectin positive cells from Liberase^{TM} digested mouse aortas.

Supplementary Information

Supplementary Tables

Supplementary Table 1

Supplementary Table1: Summary of antibodies used in this study.

Antibody	Supplier	Catalog number	Clone (if available)	Application	Working concentration
VE-cadherin	AbCAM	ab7047	BV9	Immuno-fluorescence HAoEC	2μg/ml
Claudin 5	AbCAM	ab53765	---		10μg/ml
SM22α	Proteintech	10493-1-AP	---	Immuno-fluorescence Aortic root	2μg/ml
MOMA-2	AbCAM	ab33451	---		10μg/ml
CD45-AlexaFluor 700	BioLegend	103128	30-F11	Flow cytometry mouse aortas	1μg/ml
αSMA-FITC	eBioscience	53-9760	1A4		5μg/ml
CD11b-APC	BD	561690	M1/70		1μg/ml
CD11c-PerCP-Cy5.5	BioLegend	117327	N418		2μg/ml
CD31-PE-Cy7	eBioscience	25-0311-81	390		2μg/ml
E-selectin-BV421	BD	740027	10E9.6		1μg/ml
ICAM-1-FITC	AbD Serotec	MCA1615FT	15.2	Flow cytometry HAoEC	0.25μg/1x10^6cells
VCAM-1-APC	BioLegend	305809	STA		0.25μg/1x10^6cells
E-selectin-PE	eBioscience	12-0627	P2H3		0.5μg/1x10^6cells
